# A Catalysis-Driven Dual Molecular Motor

**DOI:** 10.1021/jacs.5c01275

**Published:** 2025-03-17

**Authors:** Peng-Lai Wang, Enzo Olivieri, Stefan Borsley, George F. S. Whitehead, Avantika Hasija, David A. Leigh

**Affiliations:** † Department of Chemistry, University of Manchester, Manchester M13 9PL, U.K.; ‡ School of Chemistry and Molecular Engineering, East China Normal University, Shanghai 200062, China

## Abstract

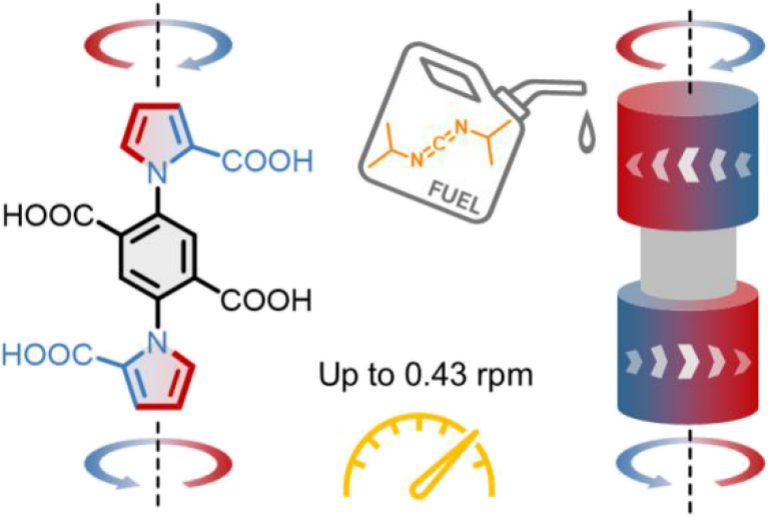

We report on a head-to-tail dual molecular motor consisting of two (identical) motor units whose pyrrole-2-carboxylic rings are turned in contra-rotary (i.e., disrotatory) fashion about a common phenyl-2,5-dicarboxylic acid stator. The motors directionally rotate via information ratchet mechanisms, in which the hydration of a carbodiimide (fuel) to form urea (waste) is catalyzed through the chemomechanical cycle of a motor unit, resulting in directional rotation about a biaryl C–N bond. The head-to-tail arrangement of the motor units produces coaxial contra-rotation of the end groups while the central phenyl ring of the axis remains dynamically unbiased. The electron-rich nature of the phenyl stator contributes to rotary catalysis by the dual-motor (and therefore motor rotation itself) being ∼7× faster than the parent 1-phenylpyrrole-2,2-dicarboxylic acid single-motor when operated under identical conditions, and 90× faster than the single-motor operated using the originally reported reaction conditions. Under batch-fueled operation (i.e., all of the fuel present at the start of motor operation), the dual-motor rotates at an initial rate of 0.43 rotations per minute (rpm). Chemostating the fuel concentration by syringe pump addition produced sustained repetitive contra-rotation at a rate of 0.24 rpm for a period of 100 min. The demonstration of chemically fueled continuous contra-rotation on a time scale of 2–4 min per rotation significantly advances the chemistry and mechanics of artificial catalysis-driven molecular machinery.

## Introduction

Many aspects of macroscopic machine design do not scale well to the molecular level.^1−3^ For example, a motor-molecule’s power output or torque cannot be increased simply by scaling up the size of the structure, as is often possible for macroscopic machinery. Instead, biology uses coupled and/or cooperative motor dynamics to enhance mechanical performance.^4^ For example, up to 11 flagella motors work together to drive the flagella,^5^ the twin motor domains of kinesin coordinate to enable the protein to ‘walk’ along microtubules,^6^ and muscle proteins operate as an ensemble to exert force.^7^ Most biomolecular machines are powered by transducing chemical energy through catalysis.^8,9^ This requires a different mechanism^10−14^ to light-driven^15−23^ molecular motors, and produces different dynamic behavior. Developing coupled and/or cooperative artificial catalysis-driven^24−31^ molecular machines offers a path to molecular nanotechnology^20,32−36^ closer to the fundamental mechanisms and dynamics of motor proteins.

**Figure 1:**
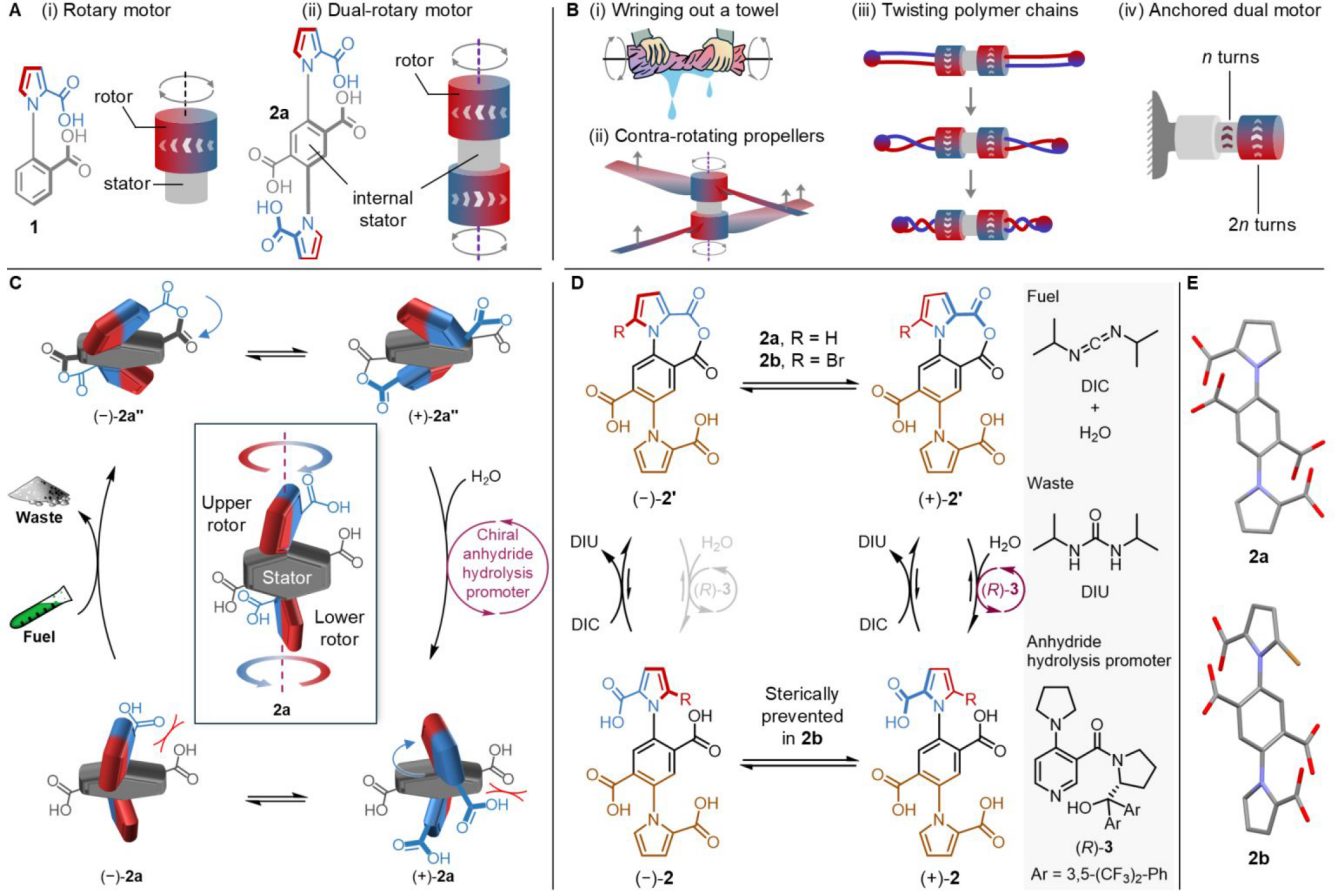
(A) Chemical structure and schematic representation of single (**1**)^28^ and dual (**2a**) rotary motor-molecules. The dual-motor has *S*_2_ symmetry, resulting in coaxial rotation of the rotors in opposite directions. (B) Examples of (i, ii) applications of coaxial contra-rotation at the macroscopic scale and (ii, iii, iv) characteristics of coaxial contra-rotation that could potentially be useful at the nanoscale. (i) Wringing out a towel is more effective by twisting the ends of the towel in opposite directions to increase the compression force.^39^ (ii) Contra-rotating propellers of opposite helicity, or rotating opposite charges in contrary directions, could generate thrust or a magnetic field, respectively. (iii) Twisting polymer strands from opposite directions could increase the force applied to contract a polymer network.^31^ (iv) Anchoring one end of a dual-motor to be stationary on a surface would, in principle, cause the other end group to directionally rotate twice as fast as a single motor unit. (C) Schematic representation of chemically powered contra-rotation of the rotors around the stator for dual-motor **2a**. For clarity, synchronized rotation of the upper and lower stator is shown, though the reaction rates indicate that the two processes are largely independent of each other. A full rotation refers to the 360° rotation of either rotor around the stator. (D) Chemomechanical cycle^8^ showing catalysis-driven rotation of the upper pyrrole ring (i.e., the top rotor; red-blue) of **2**. The lower motor unit (brown) operates through an identical mechanism. The experimentally determined reaction kinetics are consistent with the motors mechanically operating independently of each other (i.e., the rotor positions and rotations are not synchronized), although the chemical state (anhydride or diacid) of one motor may influence the rates of reaction of the other. Motor operation was carried out at concentrations where oligomerization through intermolecular anhydride formation is negligible. (E) X-ray structures obtained from single crystals of **2a** and **2b** (see Supporting Information). The structure of **2b** is disordered about the crystallographic inversion center, with Br and H at the 5-position of pyrrole having half-occupancy in the structure. C = gray, N = blue, O = red, Br = maroon. Solvents and hydrogen atoms are omitted for clarity.

Coaxial contra-rotation^37^ (i.e., rotation of two rotors in opposite directions about a common axis) of two motors on the same axle produces a distinctive set of dynamic mechanical characteristics^38,39^ (Figure 1Aii,B). In the macroscopic world, contra-rotation is often used to reduce the adverse effects of torque, leading to its widespread application in aviation and marine technologies.^38^ A similar mechanical process is produced by twisting the ends of a wet towel in opposite directions (Figure 1Bi). This exerts a greater compression force on the towel than twisting with one hand by the same amount,^39^ thus wringing out more water. In principle, contra-rotating motors^40^ could potentially prove useful at the nanoscale too, for example by increasing the extent of motor-driven contraction of a soft material (Figure 1Biii),^31^ rotating chiral propeller-shaped substituents or opposite charges in opposite directions about a common axis (Figure 1Bii), or anchoring the end of one rotor to a surface resulting in the other end rotating twice as fast (and producing twice as much power) as would an analogous single motor (Figure 1Biv).

**Figure 2:**
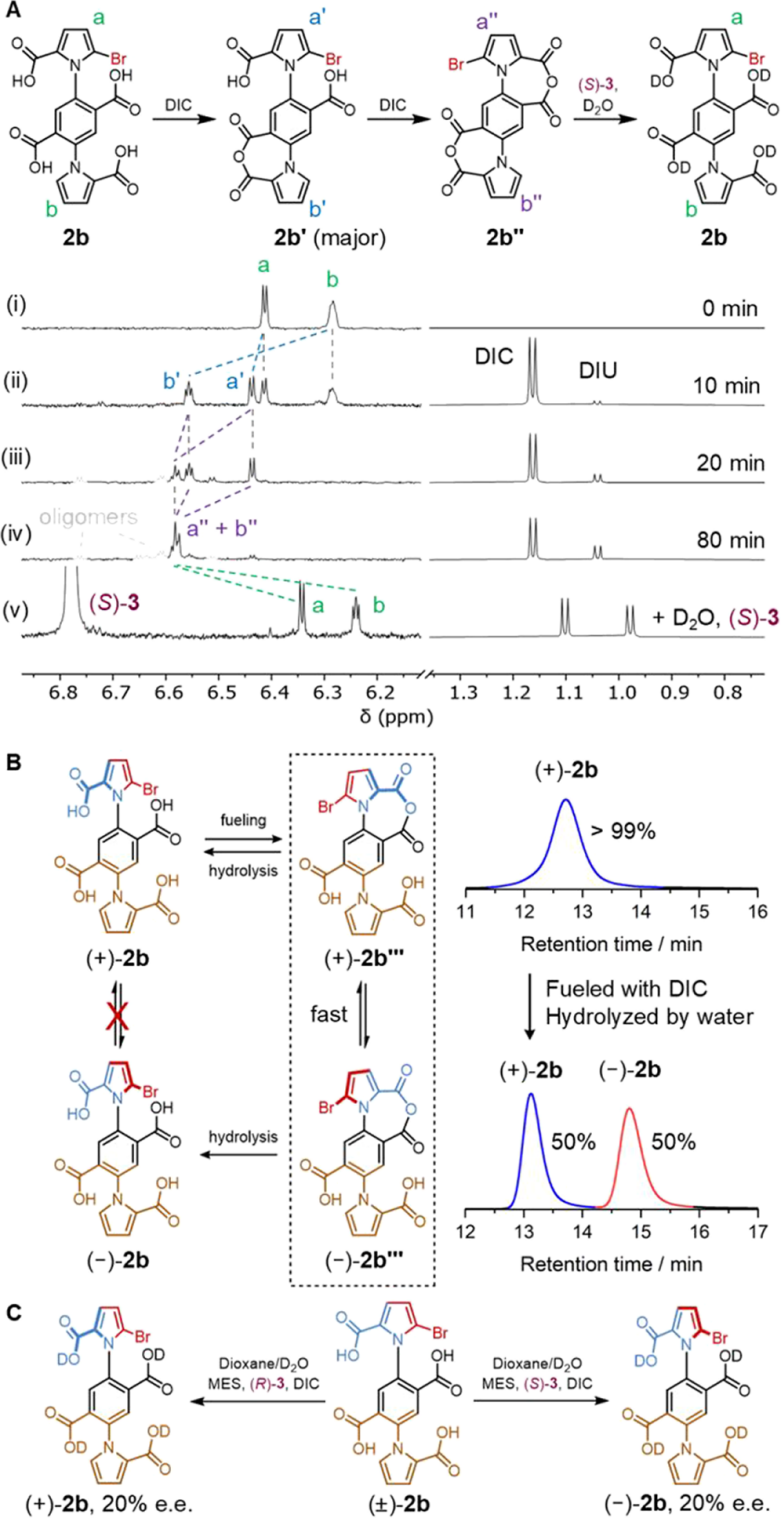
Probing the chemomechanical transitions of model dual-motor **2b**. (A) Partial ^1^H NMR spectra (dioxane-*d*_8_, 600 MHz, 298 K) of stepwise anhydride formation and hydrolysis of **2b**. The region of 6.1–6.8 ppm is scaled vertically 150× compared to the region of 0.8–1.3 ppm. (i) Motor **2b** in dioxane-*d*_8_ ([**2b**] = 0.25 mM). (ii) 10 min and (iii) 20 min (iv) 80 min after the addition of the fuel ([DIC]_0_ = 4 mM) to form **2b′** and **2b″**. (v) 5 min after the additions of hydrolysis promoter ((*S*)-**3** = 10 mM) and D_2_O (30% v/v) to reform **2b**. (B) Chiral HPLC (see Supporting Information, Section 6.2) shows the racemization of enantioenriched (±)-**2b** upon fueling with DIC ([**2b**] = 1 mM, [DIC] = 5 mM, dioxane/D_2_O (1:1 v/v)), confirming the ring-flip pathway in the chemomechanical cycle. (C) Fueling of racemic (±)-**2b** in the presence of chiral anhydride hydrolysis promoters (*R*)-**3** or (*S*)-**3** produces an equal and opposite 20% enantiomeric excess (e.e.) of the tetra-acid, demonstrating directionally biased rotation of the upper rotor about the stator in either direction ([**2b**] = 1 mM, [DIC]_0_ = 10 mM, (*R*)- or (*S*)-**3** = 10 mM, 2-(*N*-morpholino)ethanesulfonic acid (MES) monohydrate = 160 mM, pH_obs_ 5.0, dioxane/D_2_O (1:1 v/v)).

**Figure 3:**
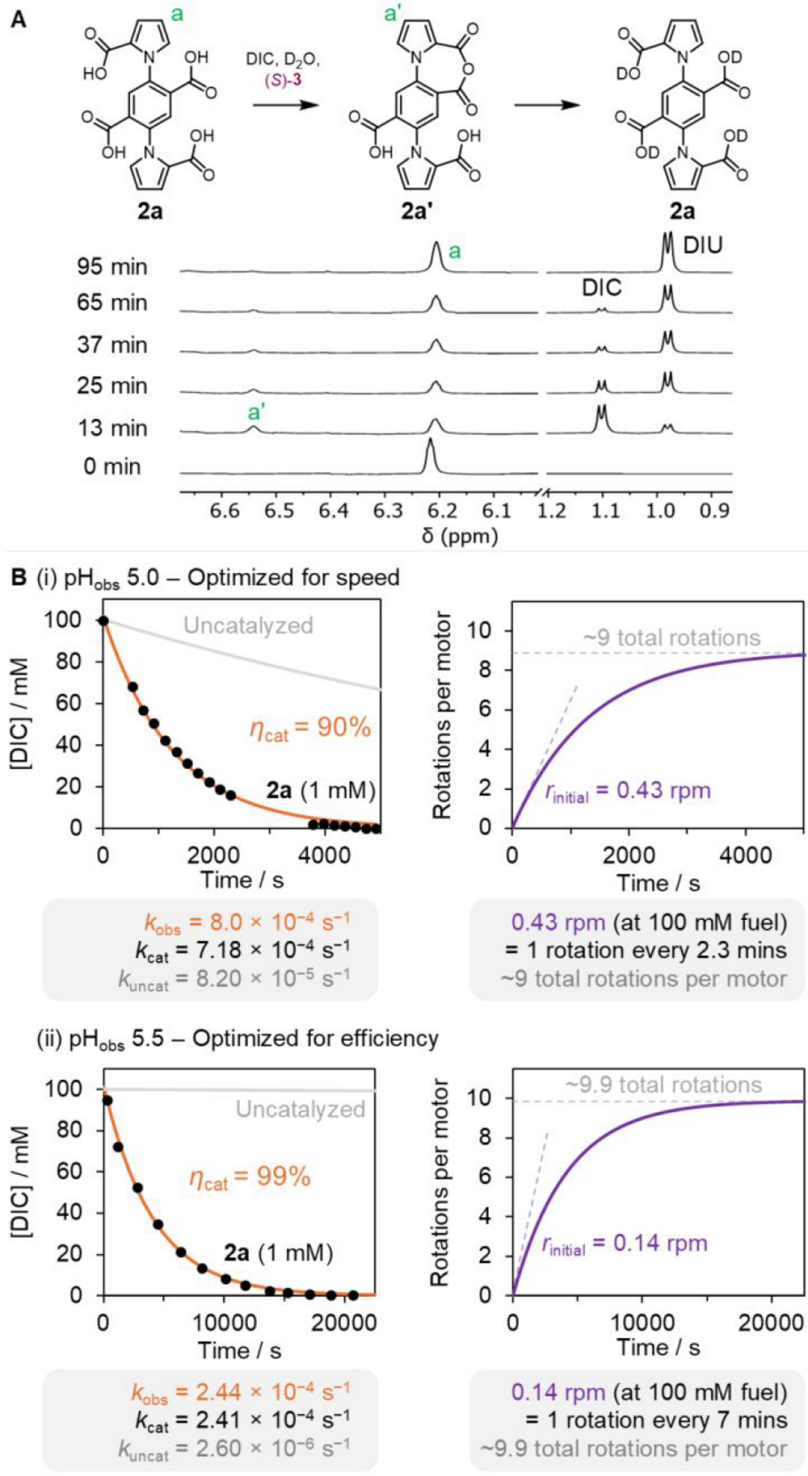
Autonomous operation of **2a**. (A) Partial ^1^H NMR spectra (dioxane-*d*_8_:D_2_O 1:1 v/v, 600 MHz, 298 K) showing the transient anhydride **2a′** formation and hydrolysis in the presence of DIC ([**2a**] = 1.0 mM, [(*S*)-**3**] = 10 mM, [DIC]_0_ = 100 mM, [MES monohydrate] = 160 mM, pH_obs_ = 5.0). The region of 6.1–6.7 ppm is scaled vertically 400× compared to the region of 0.9–1.2 ppm. (B) Kinetics of motor rotation at (i) pH of fastest rotation, pH_obs_ 5.0, and (ii) pH of greatest catalytic efficiency, pH_obs_ 5.5. Kinetics of DIC hydration in the absence and presence of **2a** (1.0 mM), determined by ^1^H NMR spectroscopy (left). Solid lines (orange and gray) represent the fit to pseudo-first-order kinetics (*k*_obs_). η_cat_ is the catalytic efficiency, i.e., the fraction of fuel that is converted to waste by a motor-mediated pathway. *r*_initial_ is the initial rotation rate. Cumulative count of rotations per motor (right, see Supporting Information, Section S6.3 for details of calculations).

**Figure 4:**
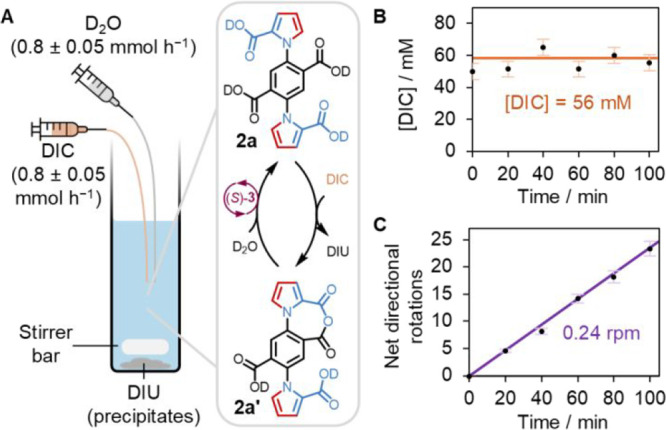
(A) Schematic representation of the autonomous operation of **2a** [5 μmol; 1 mM] under chemostated conditions by adding fuel (DIC and D_2_O) at 0.8 ± 0.05 mmol h^–1^ in the presence of chiral hydrolysis promoter (*S*)-**3** (50 μmol). The precipitation of DIU removes the waste from the solution containing the continuously operated motor. (B) Concentration of DIC as measured by ^1^H NMR spectroscopy by sampling at different time points. (C) Cumulative count of rotations per motor achieved during this operation.

**Figure 5:**
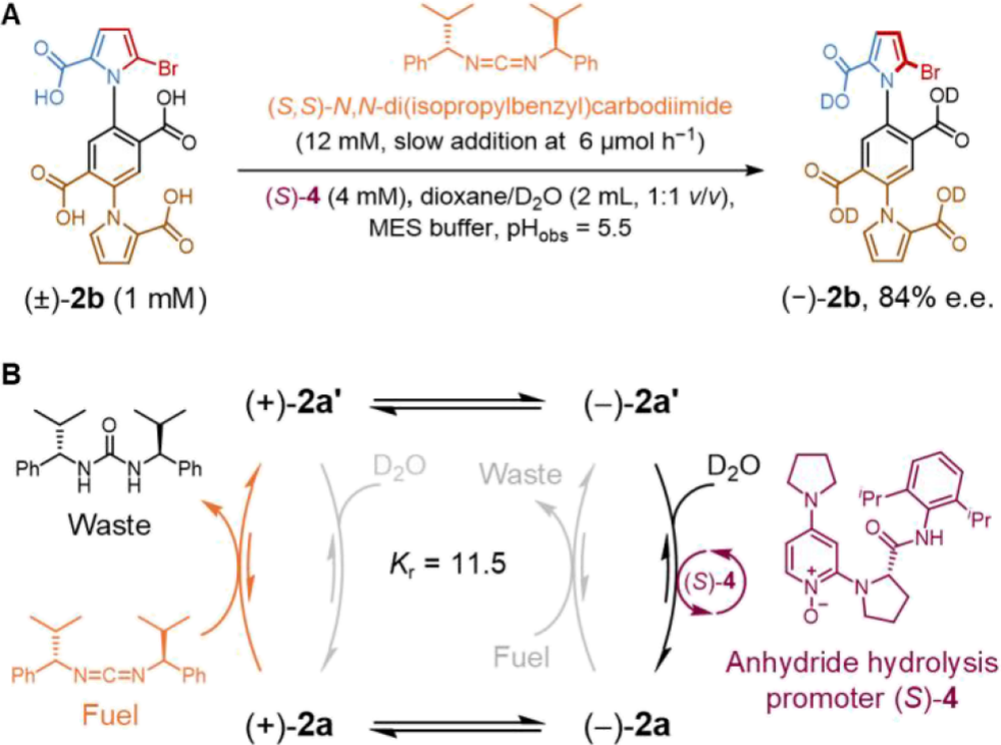
Double kinetic gating in the catalysis-driven rotation of motor **2**. (A) Racemic (±)-**2b** was fueled with a chiral fuel and chiral *N*-oxide anhydride hydrolysis promoter ([**2b**] = 1.0 mM, [(*S,S*)-*N,N*-di(isopropylbenzyl)carbodiimide] = 12.0 mM (chemostated by addition at 6 μmol h^–1^), [(*S*)-**4**] = 4.0 mM, [MES buffer] = 100 mM, pH_obs_ = 5.5, dioxane/D_2_O (2 mL, 1:1 v/v), r.t.).^45^ 84% e.e. (−)-**2b** was determined by chiral HPLC, see Supporting Information, Section S8 for details. (B) Experiments allowed evaluation of directionality resulting when both chiral fuel (*S,S*)-*N,N*-di(isopropylbenzyl)carbodiimide and chiral anhydride hydrolysis promoter (*S*)-**4** were used together.

Here we report on the design, synthesis and operation of an artificial chemically fueled,^41^ catalysis-driven,^24−31^ dual molecular motor **2a** that exhibits coaxial contra-rotation (Figure 1C). The directional bias in the individual chemical and conformational transformations were measured on a model motor **2b** in which full 360° rotation of one of the rotors is sterically blocked (Figure 2). Establishing the rates of each step in the catalytic cycle of the motor means that the rate of fuel consumption could be used to determine the rate of rotation of the dual-motor under both batch-fueled (typically all of the fuel added at once; Figure 3) and pseudo-chemostated conditions (i.e., where a constant concentration of fuel is maintained by adding fuel at the same rate it is consumed; Figures 4 and 5).

## Results and Discussion

### Design of a Contra-Rotary Dual Molecular Motor

We previously reported a 1-phenylpyrrole-2,2′-dicarboxylic acid molecular motor **1** (Figure 1Ai),^28^ where the pyrrole rotor is directionally rotated relative to the phenyl stator by the motor’s catalysis of carbodiimide-to-urea hydration.^42−44^ Although the kinetic gating for the carbodiimide addition step in the catalytic cycle is modest (∼1:1.1), the use of a chiral anhydride hydrolysis promoter (Figure 1D) causes preferential hydrolysis from one face of the motor with good enantioselectivity (2.3:1), resulting in significant kinetic asymmetry in the chemomechanical cycle.^28^ Accordingly, the motor components continuously directionally rotate about the biaryl C–N bond with directional bias during catalysis for as long as the carbodiimide fuel remains. A derivative of motor **1** was subsequently incorporated into the covalent framework of a polymer gel and the directional rotation of the motors was used to twist the polymer chains of the gel about one another, causing catalysis-driven contraction of the gel.^31^

Building on this motor design, we envisaged that a dual contra-rotary motor could be realized by incorporating two pyrrole-2-carboxylic acid units attached to the 1- and 4-positions of a phenyl-2,5-biscarboxylic acid unit, which would form an internal stator (Figure 1Aii). The direction of rotation of the dual-motor rotors is determined by the handedness of the fueling system, just as with **1**. Due to the *S*_2_ symmetry along the axle of the dual-motor, the head-to-tail rotors will rotate in opposite directions. This cancels out net torque for the central phenyl ring (the internal stator, Figure 1Aii) and means that the rotors rotate past each other twice as fast, on average, as they rotate past the internal stator (Figure 1Biv).^28^

Dual-motor **2a** (Figure 1A,C,D) and model **2b**, which features an additional bromine atom at the 5-position of one of the rotors (Figure 1D, Schemes S1 and S2), were synthesized according to procedures given in the Supporting Information (SI, Section S2). In the acid forms of **2a** and **2b** passage of each rotor carboxylic acid past the adjacent acid group on the stator is sterically blocked.^28^ However, upon fueling with a carbodiimide such as diisopropylcarbodiimide (DIC), acid **2** is converted to anhydride **2′** ((±)-**2**→**2′**; Figure 1D), which undergoes rapid interconversion of the atropisomers via a ring-flip ((−)-**2′**⇌(+)-**2′**). The chiral hydrolysis promoter (e.g., (*R*)-**3**) selectively hydrolyzes one of the tethered anhydride conformers to generate (+)-**2**. Passage of the pyrrole carboxylic acid group past the protons at the 3- and 6-positions of the phenyl unit ((+)-**2a**⇌(−)-**2a**) completes the 360° rotation of the rotor around the stator.

In compound **2b**, the steric bulk of the bromine atom prevents passage of the adjacent carboxylic acid group of the stator, leading to kinetically stable atropisomers about the upper biaryl C–N bond, allowing directionality to be assessed from the ratio of atropisomers. X-ray structures obtained from single crystals of **2a** and **2b** (obtained by slow diffusion of Et_2_O into saturated dimethylformamide and MeOH solutions of **2a** and **2b**, respectively) are structurally similar and confirm that the carboxylic acid groups on the stator cannot pass the acid groups or bromine atom on the rotors.

### Demonstrating Dual-Motor Rotation

We first confirmed that the chemical steps within the chemomechanical cycle of an individual motor, namely the conversion of acid-to-anhydride and anhydride-to-acid (Figures S4 and 2A), proceed as expected in the dual-motor. ^1^H nuclear magnetic resonance (NMR) spectroscopy showed the conversion of tetra-acid **2b** to anhydride **2b′** (the major monoanhydride) and dianhydride **2b″** upon fueling with DIC in dioxane-*d*_8_ (Figure 2A). Following the addition of 30% D_2_O and anhydride hydrolysis promoter (*S*)-**3**, the starting tetra-acid was cleanly regenerated (Figure 2Av).

We next conducted experiments to demonstrate mechanical gating,^8,14^ that is to show that the motor accesses orthogonal arcs of rotation depending on the chemical state. Due to the steric bulk of the bromine atom on the upper rotor of **2b**, the enantiomeric atropisomers (+)-**2b** and (−)-**2b** could be separated by chiral high-performance liquid chromatography (HPLC) (Figure 2B). In contrast, no atropisomers could be separated for **2a**, indicating fast passage of the pyrrole carboxylic acid over the proton at the 6-position of the phenyl group. To confirm that ring-flipping of the anhydride is a pathway that can interconvert the atropisomers, (+)-**2b** and (−)-**2b** were purified by preparative chiral HPLC and their relationship as enantiomers verified by circular dichroism (Figure S2). Treatment of (+)-**2b** (1 mM) with DIC (5 mM) in dioxane-*d*_8_:D_2_O (1:1 v/v) resulted in complete racemization within 10 min, confirming the fast dynamic interconversion between (+)-**2b‴** and (−)-**2b‴** (Figure 2B).

Having established that the motor can access the desired chemical and mechanical pathways in the reaction network, we next sought to introduce kinetic asymmetry upon fueling. Chemical gating can be introduced through enantioselective anhydride formation (by using a chiral carbodiimide fuel) and/or hydrolysis (by using a chiral anhydride hydrolysis promotor). The individual chemical gatings combine in a multiplicative manner to generate kinetic asymmetry, resulting in directional rotation.^8,11^ However, since we previously found only very modest enantioselectivity (10% e.e.) when using chiral carbodiimides for anhydride formation,^28^ we chose to operate the dual-motor with achiral DIC, thus obtaining directionality solely through the anhydride hydrolysis in a single chemically gated manner.^25^ The hydration of this less-bulky achiral fuel is catalyzed substantially faster than the bulkier chiral carbodiimides, thus potentially increasing motor speed while sacrificing only a very small degree of directionality.^27,28^

Racemic model motor **2b** (1 mM) was treated with DIC (10 mM), hydrolysis promotor (*S*)-**3** (10 mM) and MES monohydrate (160 mM) in a mixture of dioxane:D_2_O (1:1 v/v) (Figures 2C and S7). HPLC analysis of the fueled mixture indicated a 20% e.e. of (−)-**2b**, demonstrating directionally biased rotation as a consequence of chemical gating of anhydride hydrolysis. Using the opposite handedness of the hydrolysis promoter ((*R*)-**3**) afforded equal and opposite chiral induction (Figures 2C and S7). These results quantify the directionality of powered rotation of the rotor, indicating that it rotates with a modest directionality of 1.5:1, i.e., a backward rotation once every ∼ 2.5 forward turns (kinetic asymmetry, *K*_r_, = 1.5). The *S*_2_ symmetry of the motor-molecule results in the two rotors undergoing coaxial contra-rotation about the axle defined by the C–N bonds.

### Quantifying Aspects of Dual-Motor Rotation

With the directionality of the coaxial contra-rotation of the dual-motor established, we sought to quantify other key performance indicators of the motor, such as catalytic efficiency η_cat_ (i.e., the fraction of fuel that is converted to waste by a motor-mediated pathway), coupling efficiency η_rot_ (i.e., the net directional rotations per equivalent of fuel consumed by the motor), fuel efficiency η_rot/fuel_ (i.e., net directional rotations per equivalent of supplied fuel) and speed of rotation *r* (Supporting Information, Sections S6.3.4–6.3.6).^45^ To do so we first carried out autonomous motor operation under batch fueling conditions, meaning DIC (in large excess) was only added at the start of the experiment.

Motor **2a** (1 mM) was treated with DIC (∼100 mM), (*S*)-**3** (10 mM) and MES monohydrate (160 mM) in a mixture of dioxane-*d*_8_/D_2_O (1:1, v/v, pH_obs_ = 5.0, Table S1). ^1^H NMR spectroscopy showed transient formation of anhydride **2a′**, which was fully hydrolyzed to reform tetraacid **2a** once all of the DIC was consumed (∼95 min; Figure 3A). No formation of dianhydride **2a″** was observed, indicating the rapid hydrolysis of anhydride species, consistent with independent rotation of the upper and lower rotors (Figure S6). The concentration of DIC over time was plotted for both the motor-catalyzed and uncatalyzed (i.e., an experiment lacking **2a**) pathways (Figure 3B). The reaction kinetics were well-described as a pseudo-first-order process, with the motor-catalyzed rate approximately 10× greater than the uncatalyzed rate (i.e., the background rate of DIC hydration), indicating that >90% of fuel-to-waste reactions proceed by the motor-catalyzed pathway at pH 5.0 (Figure 3Bi). Raising the pH to 5.5 increased this catalytic efficiency further to 99% of fuel-to-waste reactions proceeding via the motor-catalyzed pathway, though at a slower overall rate of motor-catalysis and therefore motor rotation (Figure 3Bii, see Supporting Information, Section S6.3).

The catalysis of carbodiimide hydration by **2** occurs through a number of different chemomechanical pathways (Figure S9), namely coupled cycles (i.e., 360° directional rotation involving net interconversion of fuel and waste), futile cycles (i.e., net consumption of fuel without net movement occurring) and slip cycles (rotation without the net consumption of fuel).^14^ For further discussion of the catalysis reaction network see Supporting Information (Figure S9). In the dual-motor, as with the corresponding single-motor, slip cycles are vanishingly rare because of the high chemical potential of the fuel relative to the waste, and the large energy barrier for the acid groups to slip past each other in either chemical state of a motor unit.^28^

When converting chemical fuel to waste through rotational catalysis (*K*_r_ of 1.5), motor **2a** undergoes 50% of rotations through coupled cycles, 30% clockwise rotation (with (*R*)-**3**) and 20% counterclockwise motion. The remaining 50% of fuel correspond to hydrolysis of the same anhydride conformer as originally formed (i.e., futile fueling cycles). This gives a coupling efficiency η_rot_ of 10% (i.e., the proportion of fuel consumed by the motor that contributes to net rotation). The fuel efficiency η_rot/fuel_ is directly linked to the motor’s speed *r*, as it is the product of the coupling efficiency η_rot_ and the catalytic efficiency η_cat_ of motor **2a** (Supporting Information, Sections S6.3.5 and S6.3.6).

Although motor directionality is unaffected by changing the pH, we found that pH differences have a substantial influence on the rate of rotation of the motor. The fastest rate is achieved in the initial stages of fueling (when the fuel concentration is highest), with the two rotors turning in opposite directions at an initial rate of ∼0.215 rotations per minute (rpm) at pH_obs_ 5.0, resulting in a combined coaxial contra-rotation of ∼0.43 rpm at 100 mM fuel, i.e., a directional rotation every 2.3 min, approximately 90× faster than was previously obtained for single-motor **1** in a solvent system with a lower water content (dioxane-*d*_8_:D_2_O 7:3 v/v) at pH 5.1.^28^ We attribute this dramatic difference in rates to several factors:

1. The addition of a second phenylpyrrole dicarboxylic acid unit doubles the number of catalytic sites within the molecule.
2. The addition of a second pyrrole group to the phenyl stator makes it more electron-rich than in the single-motor, which enhances the turnover frequency of motor **2a** in the fuel-to-waste reaction. This includes a substantial increase in the rate of anhydride formation, previously identified as the rate-determining step in motor **1**.^28^
3. The 10-fold increase in the equivalents of the anhydride hydrolysis promoter (*S*)-**3** used ensures that hydrolysis proceeds efficiently, maintaining anhydride formation as the rate-determining step.
4. The use of a higher water content and lowering the pH for motor operation was found to substantially increase the hydrolysis rate at the expense of only a small increase in the background hydrolysis rate (the catalytic efficiency of motor operation was kept ≥90%).

To evaluate the influence of the structural changes, we operated the parent single-motor, **1**, under the same conditions as the dual-motor ([motor **1**] = 1.0 mM, [(*S*)-**3**] = 10 mM, [DIC]_0_ = 100 mM, [MES monohydrate] = 160 mM, dioxane-*d*_8_:D_2_O 1:1 v/v, pH_obs_ = 5.0, Supporting Information, Section S6.3.7), allowing direct comparison of the two motors. Under these conditions, single-motor **1** exhibits a catalytic efficiency of 57% (compared to 90% for dual-motor **2a**), and performs a directional rotation at an initial rate of approximately 0.06 rpm at 100 mM fuel, i.e., a directional rotation every 17 min (compared to 0.43 rpm at 100 mM for dual-motor **2a**). Thus, dual-motor **2a** rotates approximately 7× faster than single-motor **1** under identical conditions. We also note that under these conditions, motor **1** showed significant *N*-acylurea formation (Figure S14), which was not observed for dual-motor **2a**. These three factors, increased catalytic efficiency, increased rate of rotation and biproduct-free reaction are consistent with an increased nucleophilicity of motor **2a** compared to motor **1**, as a consequence of the additional pyrrole substituent on the phenyl stator.

We further probed the rotation of motor **2a** under chemostated conditions. Adding fuel by syringe pump at the same rate that it reacts, accompanied by crystallization of the DIU waste, results in a constant fuel and waste concentration (Figure 4A). Motor **2a** (1 mM), (*S*)-**3** (10 mM) and MES monohydrate (160 mM, pH_obs_ 5.0) were dissolved in dioxane/D_2_O (1:1, v/v). Continual addition of DIC and D_2_O at a rate of 0.8 ± 0.05 mmol per hour. An aliquot was taken every 20 min to monitor the constant concentration of DIC by ^1^H NMR spectroscopy, which indicated the system is efficiently chemostated ([DIC] = 56 mM), the motor concentration remains constant at 1 mM, and the presence of urea waste (which precipitates from the reaction medium) does not affect the rate of any of the other processes (see Supporting Information Figure S16).

The chemostated experiment at this fuel concentration corresponds to a sustained rotation rate for each motor **2a** of 0.24 rpm (or 1 rotation every 4 min) (Figure 4C). No side products nor degradation of the motor was detected following the extended operation of the motor (>200 catalytic cycles; >20 net directional rotations). Note that the rate of catalysis does not change over time in Figure 4C. As the fastest rate of catalysis occurs by directional progression around the catalytic cycle, the unchanging rate of catalysis provides direct experimental evidence that the motor-molecule components are continuously directionally rotating. The experiment demonstrates that artificial molecular motors can undergo sustained autonomous operation under chemostated conditions.

We also investigated fueling motor **2** under conditions optimized for directionality at the expense of rotation rate (Figure 5).^45^ Compound **2b** (1 mM) was treated with (*S,S*)-*N,N*-di(isopropylbenzyl)carbodiimide (12 mM), hydrolysis promoter (*S*)-**4** (4 mM), and MES buffer (100 mM) in a mixture of dioxane/D_2_O (1:1, v/v, pH_obs_ = 5.5) (Figure 5A). HPLC analysis of the fueled mixture indicated an 84% e.e. of (−)-**2b**, as a consequence of the double kinetic gating^25^ and improved enantioselectivity of hydrolysis promoter (*S*)-**4**.^45^ This corresponds to a directionality of 11.5 forward rotations per backward rotation (*K*_r_ = 11.5)^14^ (Figures 5B and S17).

Under conditions optimized for rotation rate, the initial rate of 0.43 rpm (all fuel added at the start), and sustained rate of 0.24 rpm over 100 min (fuel continuously added by syringe pump) make dual-motor **2a** by far the fastest synthetic catalysis-driven small-molecule motor reported to date.^24,28,30,46^ However, it is much slower than many biomolecular motors, which typically rotate at 60–10,000 rpm.^47^ Some generations of Feringa-type light-driven motors have the theoretical potential to rotate at MHz, or even GHz, frequencies if the rate-determining step in the motor operation is thermal helix inversion.^48^ However, with conventional (i.e., nonlaser) light sources their speed is typically limited by the lack of photons, modest absorption and futile cycling of the motors, as well as reported^49^ frequently compromised photostability. The ‘real life’ rate of rotation of such motors is 10s of minutes per half rotation in most of the experiments reported to date.^50,51^

## Conclusions

Motor-molecule **2a** is a dual-motor-molecule that undergoes continuous and autonomous coaxial contra-rotation through an information ratchet^2,12,14,52−54^ mechanism during its catalysis of carbodiimide hydration. The structure of the dual-motor leads to a substantial improvement in the speed of chemically fueled rotation compared to the parent single-motor. Under operating conditions optimized for the speed of rotation, which produce modest directionality and fuel efficiency (one net directional rotation for every 10 fuel molecules consumed; comparable to a quantum yield of 10% for a photochemical process), the dual-motor achieves a maximum speed of rotation of one directional turn every 2 min in batch-fueled experiments. The dual-motor rotates directionally with a sustained speed of a turn every ∼4 min for 100 min under continuous fueling, with no detectable motor degradation over the operating period. This is still orders of magnitude slower than biomolecular motors but the fastest sustained speed of rotation measured to date for artificial small-molecule motors.^24,28,30,46^ Under operating conditions optimized for directionality (dilute fuel concentration), the double motor produces >11 forward rotations for each backward rotation, but under these fueling conditions the motor takes several hours per rotation. The cooperative action of motor units in designs such as **2** holds promise for various nanoscale mechanical applications, such as those shown in Figure 1Bii–iv.

## Supporting Information Available

Experimental methods and operations data and X-ray crystal structure determination method and data (PDF)
