# Supplementary material for: A Catalysis-Driven Dual Molecular Motor
Source: J Am Chem Soc. 2025 Mar 17;147(12):10690–7. doi: 10.1021/jacs.5c01275 (PMC11951142; doi:10.1021/jacs.5c01275)
Supplement: Supplementary file 1 — ja5c01275_si_001.pdf [file ja5c01275_si_001.pdf]

A catalysis-driven dual molecular motor

Peng-Lai Wang,[1] Enzo Olivieri,[1] Stefan Borsley,[1] George F. S. Whitehead,[1] Avantika Hasija,[1] David A. Leigh*[1,2]

[1] Department of Chemistry, University of Manchester, Oxford Road, Manchester M13 9PL, UK

[2] School of Chemistry and Molecular Engineering, East China Normal University, 200062 Shanghai, China

*Corresponding author. E-mail: david.leigh@manchester.ac.uk

- Supplemental Information –

Contents

S1. General methods and abbreviations 2
S2. Synthesis and characterization.... 4
S2.1 Synthesis of motor **2a**.... 4
S2.2 Synthesis of compound **2b**.... 6
S3. Chiral HPLC analysis of **2a** and **2b** 9
S4. CD spectra 10
S5. Scheme of chemical transitions.... 11
S6. Motor operations 12
S6.1 Stepwise operation 12
S6.1.1 Procedure for stepwise operation 12
S6.1.2 ^{1}H NMR spectra of stepwise operation.... 12
S6.2 Chemically driven racemization of (+)**-2b** and (−)-**2b**.... 13
S6.3 Autonomous operation 14
S6.3.1 Procedure for autonomous operation 14
S6.3.2 Chiral HPLC traces analysis of the autonomous operation of **2b**.... 15
S6.3.3 Monitoring autonomous operation kinetics by ^{1}H NMR.... 15
S6.3.4 Calculation of the coupling efficiency η_{rot} 16
S6.3.5 Fuel consumption efficiency η_{fuel} and fuel efficiency $\eta_{\mathrm{rot/fuel}}$.... 17
S6.3.6 Calculation of the rotation rate 20
S6.3.7 Operation of single motor **1**.... 23
S6.4 Consistency of pH during fuelling 25
S7. Operation of **2a** under chemostated conditions 26
S8. Operation of dual motor **2** under conditions optimized for high directionality 26
S9. Crystallographic data 27
S9.1 SCXRD experimental details.... 27
S9.2 Crystal structure determination and refinements.... 27
S10. NMR spectra 31
S11. References.... 37

S1. General methods and abbreviations

Unless stated otherwise, reagents were obtained from commercial sources and used without purification. Anhydrous solvents THF, DMF and CH_2Cl_2 were obtained by passing the solvent through an activated alumina column on a Phoenix SDS (solvent drying system; JC Meyer Solvent Systems, CA, USA). ^{1}H NMR spectra were recorded on a Bruker Avance III instrument with an Oxford AS600 magnet equipped with a cryoprobe [5mm CPDCH ^{13}C-^{1}H/D] (600 MHz). Chemical shifts are reported in parts per million (ppm) relative to tetramethylsilane from high to low frequency using the residual solvent peak as the internal reference (CD_3CN = 1.94 ppm, 1,4-dioxane-d_8 = 3.53 ppm, $(CD_3)_2SO$ =2.50 ppm). All ^{1}H resonances are reported to the nearest 0.01 ppm. The multiplicity of ^{1}H signals are indicated as: s = singlet; d = doublet; t = triplet; q = quartet; multiplet; br = broad; or combinations of thereof. Coupling constants (*J*) are quoted in Hz and reported to the nearest 0.1 Hz. Where appropriate, averages of the signals from peaks displaying multiplicity were used to calculate the value of the coupling constant. ^{13}C NMR spectra were recorded on the same spectrometer at 295 K with the central resonance of the solvent peak as the internal reference (CD_3CN = 118.26 ppm, $(CD_3)_2SO$ =39.52 ppm). All ^{13}C resonances are reported to the nearest 0.1 ppm. DEPT, COSY, HSQC and HMBC experiments were used to aid structural determination and spectral assignment. Flash column chromatography was carried out using Silica 60 Å (particle size 40–63 µm, Sigma Aldrich, UK) as the stationary phase. Preparative TLC was performed using PLC 20 × 20 cm, 60 F254 preparatory plates of various thicknesses (250–2000 µm). Analytical TLC was performed on precoated silica gel plates (0.25 mm thick, 60 F254, Merck, Germany) and visualized using both short and long wave ultraviolet light in combination with standard laboratory stains (acidic potassium permanganate, iodine vapor). Low resolution ESI mass spectrometry was performed with a Thermo Scientific LCQ Fleet Ion Trap Mass Spectrometer or an Agilent Technologies 1200 LC system with an Advion Expression CMS L single quadrupole MS detector. High-resolution mass spectrometry (HRMS) was carried out at the Mass Spectrometry Service, Department of Chemistry, University of Manchester. Compounds **S1**,[S1] **S2**[S2] and **S5**[S3] (*S*)-**4**[S4] were prepared in accordance with literature procedures.

Abbreviations: CD, Circular dichroism; DIC: diisopropylcarbodiimide; DIU: diisopropylurea; DMSO: dimethylsulfoxide; EtOAc: ethyl acetate; ESI: electrospray ionization; h: hour; HRMS: high-resolution mass spectrometry; MES: 2-

morpholinoethanesulfonic acid; NMR: nuclear magnetic resonance; ppm: parts per million; r.t.: room temperature; TFA: trifluoroacetic acid; TLC: thin layer chromatography; THF: tetrahydrofuran.

S2. Synthesis and characterization

S2.1 Synthesis of motor 2a

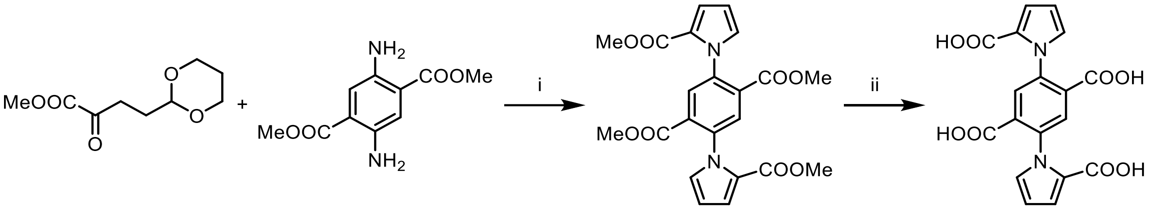

S1 **S2** **S3** **2a**

Scheme S1. Reagents and conditions for the synthesis of motor **2a**. Compounds **S1** and **S2** were synthesized according to reported procedures.[S1, S2] (i) *p*-toluenesulfonic acid monohydrate, MeOH, 80 °C, 24 h, 73%. (ii) NaOH, THF/EtOH/H_2O, 80 °C, 36 h, 90%.

S3

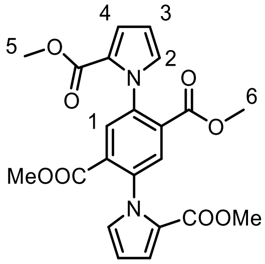

To a Schlenk tube were added **S1** (303 mg, 1.5 mmol), **S2** (112 mg, 0.5 mmol), *p*-toluenesulfonic acid monohydrate (190 mg, 1.0 mmol) and methanol (8.0 mL). The mixture was heated to 80 °C for 24 h. The mixture was diluted with EtOAc (20 mL) and washed with water and brine. The organic phase was dried over anhydrous sodium sulfate and the solvent was removed under reduced pressure. The crude product was purified through flash column chromatography (SiO_2) eluting with petroleum ether/EtOAc (4:1→2:1, *v*/*v*) to afford **S3** (161 mg, 73%) as a yellow solid.

^{1}H NMR (600 MHz, $(CD_3)_2SO$) δ 7.84 (s, 2H, H_1), 7.33 – 7.20 (m, 2H, H_2), 7.05 (s, 2H, H_4), 6.37 (s, 2H, H_3), 3.61 (s, 12H, $H_{5,6}$).

^{13}C NMR (151 MHz, $(CD_3)_2SO$) δ 163.6, 160.1, 138.9, 131.5, 130.4, 130.2, 123.7, 123.6, 118.1, 109.8, 52.5, 51.1.

HRMS (ESI$^+$) calcd. For $C_{22}H_{20}N_2O_8Na$ $[M+Na]^+$ = 463.1112, found 463.1123.

2a

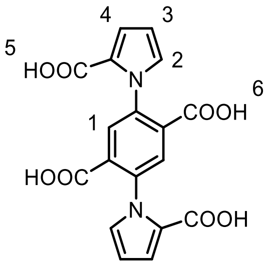

NaOH (240 mg, 6.0 mmol) was added to the stirred solution of compound **S3** (176 mg, 0.4 mmol) in a mixture of THF, ethanol and water (12 mL, 1:1:1 *v*/*v*/*v*). The solution was heated to 80 °C and stirred for 36 hours. The mixture was cooled to room temperature, acidified to pH = 3 with 2 M HCl, and extracted with EtOAc (20 mL). The organic phase was dried over anhydrous sodium sulfate and the solvent was removed under reduced pressure. The crude product was washed with diethyl ether to afford **2a** (138 mg, 90%) as a yellow solid.

^{1}H NMR (600 MHz, $(CD_3)_2SO$) δ 13.20 (s, 2H, H_6), 12.13 (s, 2H, H_5), 7.71 (s, 2H, H_1), 7.19 – 7.11 (m, 2H, H_2), 6.96 (s, 2H, H_4), 6.30 (s, 2H, H_3).

^{13}C NMR (151 MHz, $(CD_3)_2SO$) δ 165.0, 161.0, 138.8, 132.3, 130.1, 129.8, 124.8, 117.9, 109.2.

HRMS (ESI^+) calcd. For $C_{18}H_{11}N_2O_8$ $[M-H]^-$ = 383.0521, found 383.0522.

S2.2 Synthesis of compound 2b

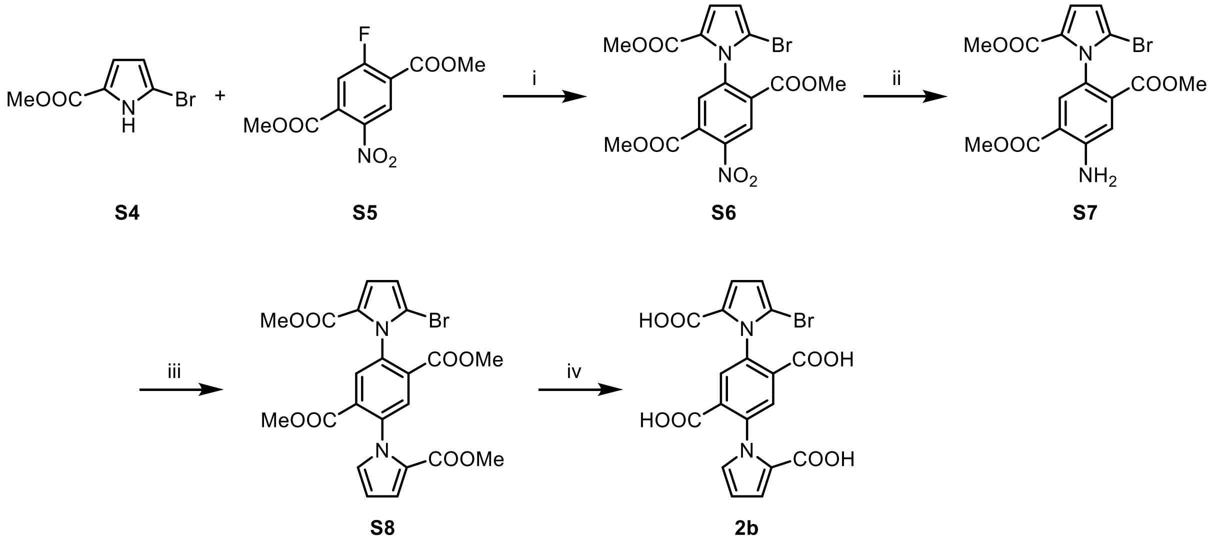

Scheme S2. Reagents and conditions for the synthesis of compound **2b**. Compounds **S5** were synthesized according to reported procedures.[S3] (i) K_2CO_3, DMF, 80 °C, 24 h, 45%. (ii) Zn powder, HCl, MeOH, 25 °C, 24 h, 74%. (iii) **S1**, *p*-toluenesulfonic acid monohydrate, MeOH, 80 °C, 24 h, 70%. (iv) NaOH, THF/EtOH/H_2O, 80 °C, 36 h, 87%.

S6

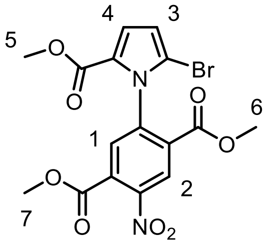

To a Schlenk tube were added **S4** (122 mg, 0.6 mmol), **S5** (128 mg, 0.5 mmol), K_2CO_3 (276 mg, 2.0 mmol) and DMF (5.0 mL). The mixture was heated to 80 °C for 24 hours. The mixture was diluted with EtOAc (15 mL) and washed with water and brine. The organic phase was dried over anhydrous sodium sulfate and the solvent was removed under reduced pressure. The crude product was purified through flash column chromatography (SiO_2) eluting with petroleum ether/EtOAc (5:1, *v*/*v*) to afford **S6** (99 mg, 45%) as a colorless solid.

^{1}H NMR (600 MHz, CD_3CN) δ 8.57 (s, 1H, H_2), 7.79 (s, 1H, H_1), 7.07 (d, J = 4.1 Hz, 1H, H_4), 6.50 (d, J = 4.1 Hz, 1H, H_3), 3.91 (s, 3H, H_7), 3.71 (s, 3H, H_6), 3.63 (s, 3H, H_5).

^{13}C NMR (151 MHz, CD_3CN) δ 164.9, 163.4, 160.6, 148.1, 143.2, 133.7, 133.1, 131.3, 127.6, 126.8, 119.5, 113.8, 112.1, 54.4, 53.8, 52.1.

HRMS (ESI$^+$) calcd. For $C_{16}H_{13}BrN_2O_8Na$ $[M+Na]^+$ = 462.9747, found 462.9754.

S7

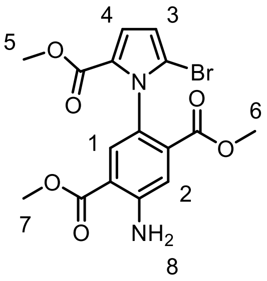

To a round-bottom flask were added **S6** (110 mg, 0.25 mmol), Zn powder (65 mg, 1.0 mmol), acetic acid (3.0 mL) and ethyl acetate (3.0 mL). The mixture was stirred at room temperature for 24 hours. The mixture was diluted with EtOAc (50 mL)and washed with water and brine. The organic phase was dried over anhydrous sodium sulfate and the solvent was removed under reduced pressure. The crude product was purified through flash column chromatography (SiO_2) eluting with petroleum ether/EtOAc (4:1, *v*/*v*) to afford **S7** (76 mg, 74%) as a colorless solid.

^{1}H NMR (600 MHz, $(CD_3)_2SO$) δ 7.63 (s, 1H, H_1), 7.38 (s, 1H, H_2), 6.98 (d, J = 4.1 Hz, 1H, H_4), 6.40 (s, 2H, H_8), 6.37 (d, J = 4.1 Hz, 1H, H_3), 3.83 (s, 3H, H_6), 3.61 (s, 3H, H_7), 3.60 (s, 3H, H_5).

^{13}C NMR (151 MHz, $(CD_3)_2SO$) δ 168.0, 165.5, 160.7, 151.9, 134.7, 133.5, 126.6, 126.2, 119.4, 118.7, 113.8, 112.6, 112.3, 53.0, 52.6, 51.6.

HRMS (ESI^+) calcd. For $C_{16}H_{15}BrN_2O_6Na$ $[M+Na]^+$ = 433.0006, found 433.0016.

S8

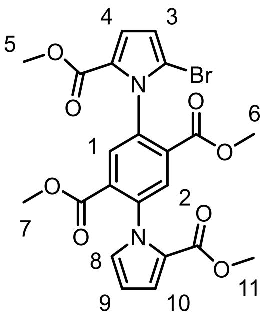

To a Schlenk tube were added **S1** (40 mg, 0.2 mmol), **S7** (70 mg, 0.17 mmol), *p*-toluenesulfonic acid monohydrate (32 mg, 0.17 mmol) and methanol (4.0 mL). The mixture was heated to 80 °C for 24 h. The mixture was diluted with EtOAc (10 mL) and washed with water and brine. The organic phase was dried over anhydrous sodium sulfate and the solvent was removed under reduced pressure. The crude product was

purified through flash column chromatography (SiO_2) eluting with petroleum ether/EtOAc (4:1→2:1, *v*/*v*) to afford **S8** (62 mg, 70%) as a yellow solid.

^{1}H NMR (600 MHz, $(CD_3)_2SO$) δ 7.95 (s, H, H_1), 7.85 (s, 1H, H_2), 7.32 (s, 1H, H_8), 7.10 (s, 1H, H_4), 7.06 (s, 1H, H_{10}), 6.57 (s, 1H, H_3), 6.38 (s, 1H, H_9), 3.64 – 3.59 (m, 12H, $H_{5,6,7,11}$).

^{13}C NMR (151 MHz, $(CD_3)_2SO$) δ 163.6, 163.0, 160.0, 159.2, 159.1, 139.8, 137.4, 132.0, 131.9, 130.7, 130.6, 130.3, 125.3, 125.1, 123.9, 123.7, 118.5, 118.4, 118.3, 112.4, 112.1, 111.8, 109.9, 52.8, 52.6, 51.3, 51.1, 51.1.

HRMS (ESI$^+$) calcd. For $C_{22}H_{19}BrN_2O_8Na$ $[M+Na]^+$ = 541.0217, found 541.0237.

2b

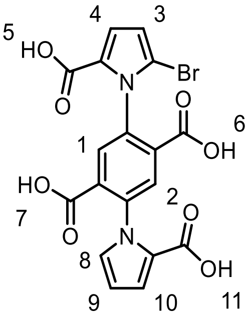

NaOH (120 mg, 3.0 mmol) was added to the stirred solution of compound **S8** (104 mg, 0.2 mmol) in a mixture of THF, ethanol and water (6.0 mL, 1:1:1 *v*/*v*/*v*). The solution was heated to 80 °C and stirred for 36 h. The mixture was cooled to room temperature, acidified to pH = 3 with 2 M HCl, and extracted with EtOAc (10 mL). The organic phase was dried over anhydrous sodium sulfate and evaporated under reduced pressure. The crude product was washed with diethyl ether to afford **2b** (80 mg, 87%) as a yellow solid.

^{1}H NMR (600 MHz, $(CD_3)_2SO$) δ 13.28 (s, 2H, $H_{6,7}$), 12.29 (s, 1H, H_5), 12.13 (s, 1H, H_{11}), 7.81 (s, H, H_1), 7.67 (s, 1H, H_2), 7.19 (s, 1H, H_8), 7.01 (d, J = 4.0 Hz, 1H, H_4), 6.97 (s, 1H, H_{10}), 6.49 (d, J = 4.0 Hz, 1H, H_3), 6.31 (s, 1H, H_9).

^{13}C NMR (151 MHz, $(CD_3)_2SO$) δ 164.9, 164.4, 161.0, 160.0, 139.8, 137.3, 132.7, 131.9, 130.6, 129.8, 129.6, 126.3, 124.9, 118.0, 111.9, 117.9, 111.1, 109.3.

HRMS (ESI$^+$) calcd. For $C_{18}H_{10}BrN_2O_8$ $[M-H]^-$ = 460.9626, found 460.9608.

S3. Chiral HPLC analysis of 2a and 2b

To analyze **2a** and **2b,** Chiral stationary phase high-performance liquid chromatography (chiral HPLC) analysis was performed (Figure S1) on a diacel ChiralPak IA column (4.6 mm × 25 mm, 5 µm particle size). A mixture of *i*-PrOH:*n*-hexane:0.1% TFA in CH_2Cl_2 (7:88:5 *v*/*v*/*v*) was used as an eluent at 25 °C with a flow rate of 1 mL min^{-1}. Traces based on the absorbance at 254 nm are reported.

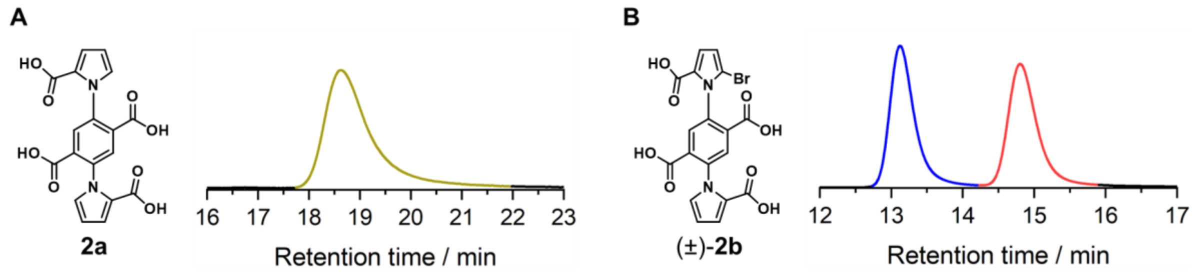

Figure S1. Chiral HPLC analysis of **2a** and **2b**. Conditions: ChiralPak IA column (4.6 mm × 25 mm, 5 µm particle size), 25 °C, *i*-PrOH:*n*-hexane:0.1% TFA in CH_2Cl_2 (7:88:5 *v*/*v*/*v*), 1 mL min^{-1}. A) Chemical structure (left) and chiral HPLC trace (right) of **2a**. No atropisomers were observed but a single peak, highlighted in golden. B) Chemical structure (left) and chiral HPLC trace (right) of **2b**. Two atropisomeric peaks were observed as highlighted in blue and red.

S4. CD spectra

Circular dichroism (CD) and ultraviolet-visible (UV-Vis) spectroscopy were measured on an Applied Photophysics Ltd Chirascan CD Spectrometer using 2 mm cuvettes at room temperature. Samples were prepared in dioxane/D_2O (7:3 *v*/*v*).

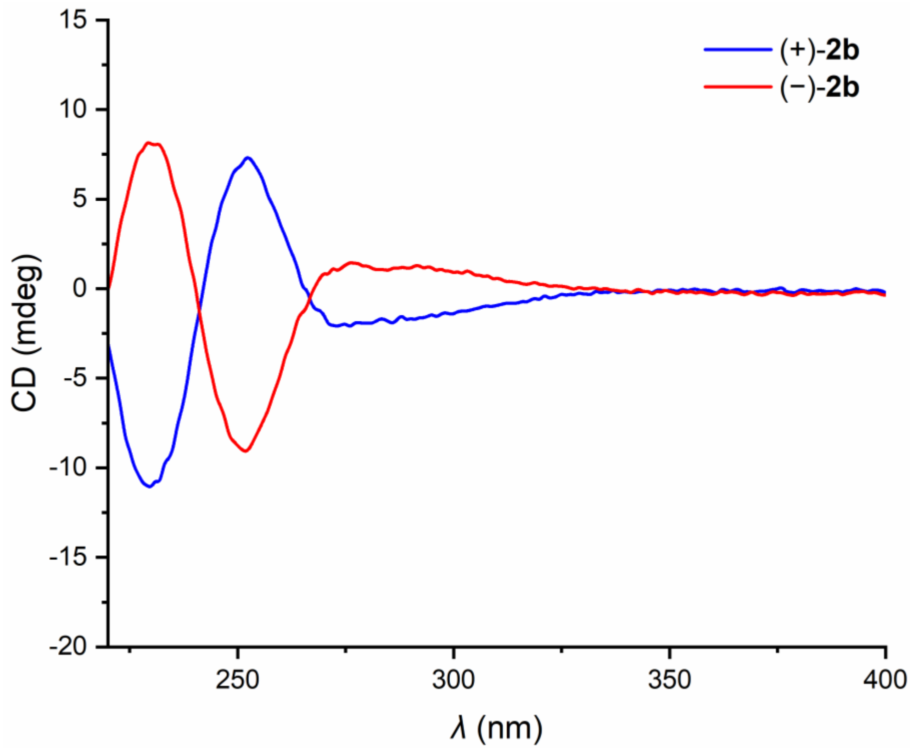

Figure S2. CD spectra of enantiomers (+)-**2b** (blue) and (−)-**2b** (red) in dioxane/D_2O (7:3 *v*/*v*) ([**2b**] ≈ 0.5 mM).

S5. Scheme of chemical transitions

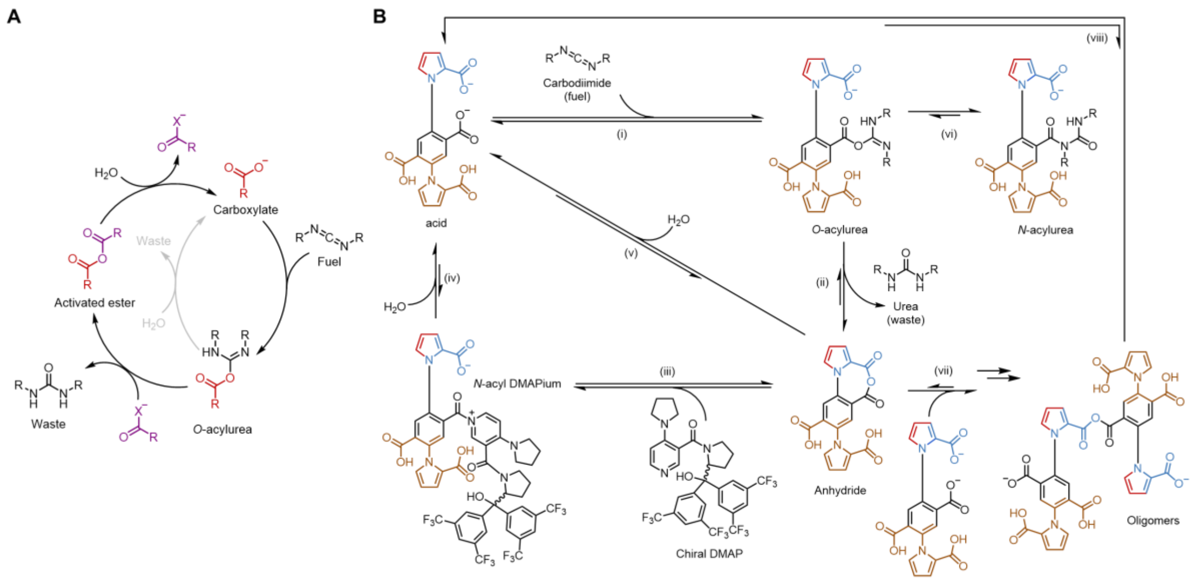

Figure S3 A) Catalytic cycle for the hydration of a carbodiimide using a carboxylate catalyst. B) Expanded chemical scheme showing the most important transitions of the motor under fuelling conditions. Only reaction of the upper pyrrole ring of the motor is shown. The lower motor unit (brown) operates through an identical mechanism. All chirality is omitted for clarity. All arrows are shown as equilibria to be thermodynamically consistent, though equilibria shown as non-equivalent are effectively irreversible under the operation conditions. (i) Reaction of the motor with carbodiimide generates *O*-acylurea. Only reaction of the bottom, more electron rich carboxylate is shown, though in principle either carboxylate may react. (ii) Nucleophilic attack of the *O*-acylurea to form the cyclic anhydride. (iii) Ring opening of the anhydride with the cyclic anhydride with a nucleophilic anhydride hydrolysis catalyst. Only reaction of the bottom carboxylate is shown, though in principle either carboxylate may react. (iv) Hydrolysis of the adduct of the motor and hydrolysis catalyst to regenerate the diacid. (vi) Rearrangement of the *O*-acylurea to the unreactive *N*-acylurea. Due to the very fast intramolecular attack of the *O*-acylurea to form the anhydride, this unproductive *N*-acylurea formation is not observed under the reaction conditions. (vii) Ring opening of the cyclic anhydride with another molecule of diacid motor can lead to formation of intermolecular anhydrides. Further activation of the carboxylates and intermolecular reaction can lead to the formation of longer oligomers. (viii) Hydrolysis of the intermolecular anhydrides regenerates the diacid motor. This process may occur directly or involve the hydrolysis catalyst (not shown).

S6. Motor operations

S6.1 Stepwise operation

S6.1.1 Procedure for stepwise operation

[**2a**] = 1.0 mM, [(*S*)-**3**] = 10 mM, [DIC] = 2.0 mM, [MES monohydrate] = 160 mM in dioxane-d_8:D_2O (1:1 *v*/*v*).

2a (1.0 µL of a 1.0 M stock solution) was dissolved in dioxane-d_8 (500 µL) and a ^{1}H NMR spectrum was obtained. DIC (2.0 µL of a 1.0 M stock solution, 2.0 mM) was added and the reaction was monitored by ^{1}H NMR spectroscopy until anhydride **2a''** formation had gone to completion. (*S*)-**3** (10 µmol), MES monohydrate (160 µmol) and D_2O (500 µL) were added, and a ^{1}H NMR spectrum was obtained. A ^{1}H NMR spectrum was recorded to confirm complete hydrolysis to tetraacid **2a**.

S6.1.2 ^{1}H NMR spectra of stepwise operation

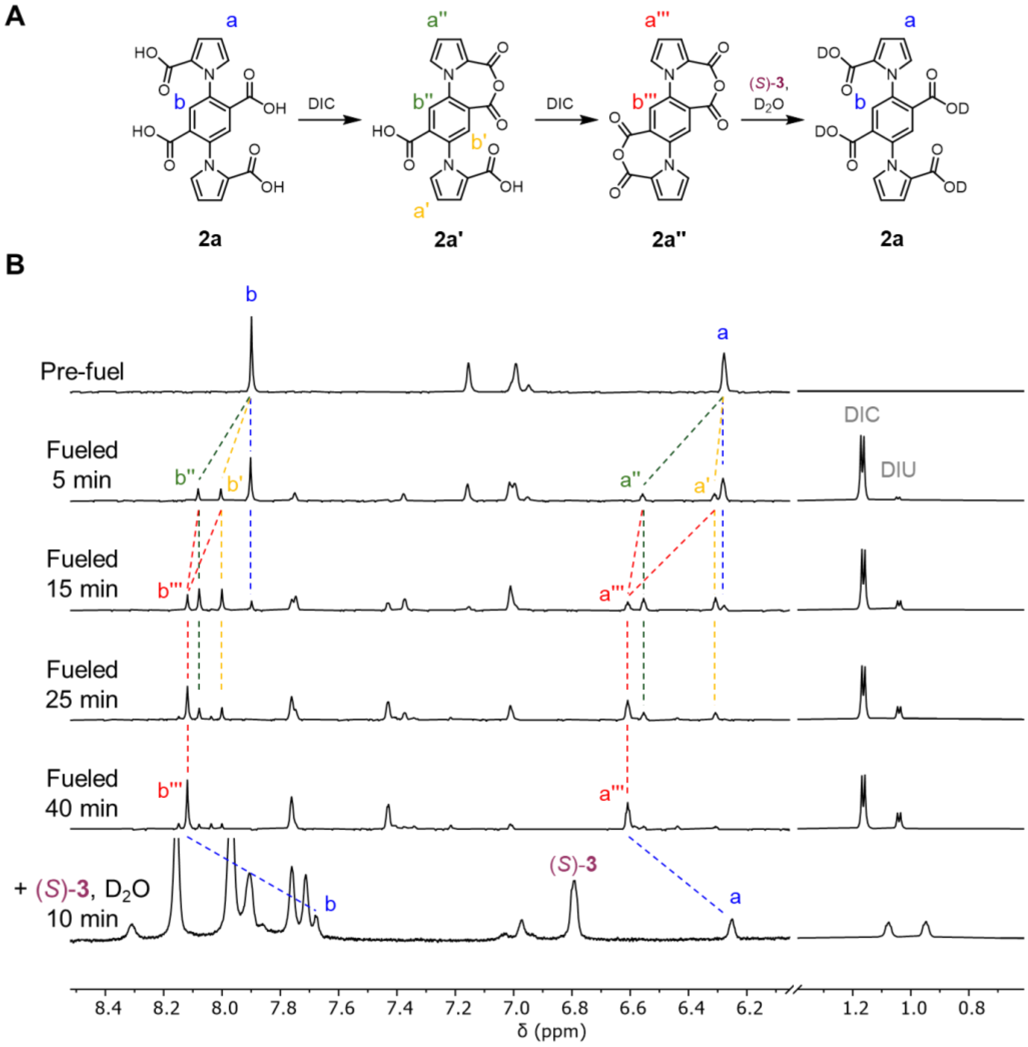

Figure S4. A) Stepwise anhydride formation and hydrolysis of motor **2a**. B) Partial ^{1}H NMR spectra (dioxane-d_8, 600 MHz, 298 K) of stepwise operation. The region 6.1–8.5 ppm is scaled vertically 30×

compared to region 0.6–1.4 ppm. Motor **2a** in dioxane-*d*8 ([**2a**] = 0.5 mM) was fueled with carbodiimide fuel (5 eq., [DIC] = 2.5 mM), which reacts with motor **2a** to form anhydride **2a'** and urea waste. Addition of chiral anhydride hydrolysis promoter (*S*)-**3** (10 eq., [(*S*)-**3**] = 5 mM) and MES monohydrate (160 eq., [MES monohydrate] = 80 mM) and D_2O (50% *v*/*v*), results in hydrolysis of anhydride **2a'**, reforming motor **2a**.

S6.2 Chemically driven racemization of (+)-2b and (−)-2b

Atropisomers of **2b** were separated by chiral HPLC (ChiralPak IA column (4.6 mm × 25 mm, 5 µm particle size), 25 °C, *i*-PrOH:*n*-hexane:0.1%TFA in CH_2Cl_2 (7:88:5 *v*/*v*/*v*), 1 mL min^{-1}), the left peak was labelled (highlighted in blue) as (+)-**2b**, and the right peak (highlighted in red) as (−)-**2b** (see Figure S5). Pure, separated samples of (+)- and (−)-**2b** were dissolved in dioxane ([**2b**] = 1.0 mM) and were operated according to the general procedure given in S6.3.1. HPLC analysis of the operated samples revealed complete racemization (see Figure S5).

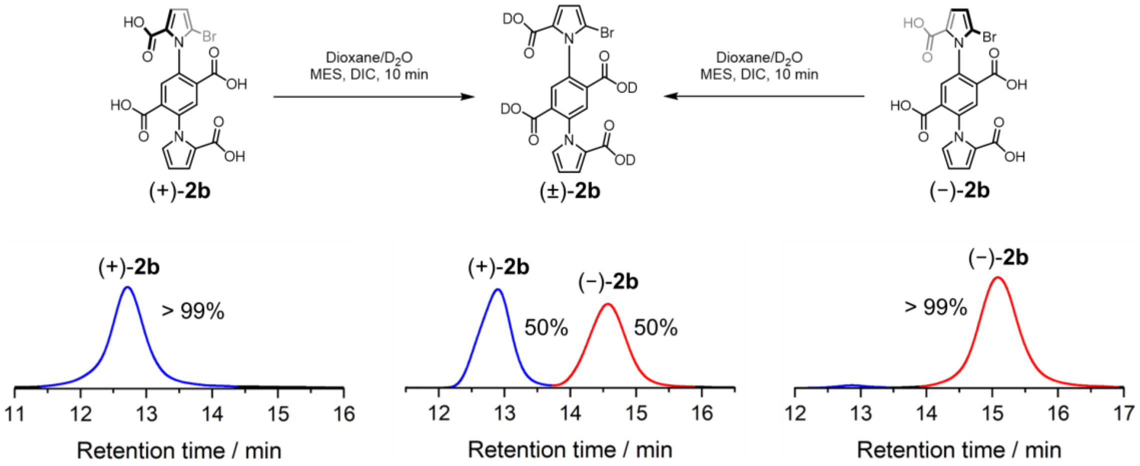

Figure S5. Chemically driven racemization of separated enantiomers of **2b**. Treatment of chiral HPLC separated enantiomers of **2b** with DIC and D_2O (top) resulted in complete racemization as determined by chiral HPLC (bottom, ChiralPak IA column (4.6 mm × 25 mm, 5 µm particle size), 25 °C, *i*-PrOH:*n*-hexane:0.1% TFA in CH_2Cl_2 (7:88:5 *v*/*v*/*v*), 1 mL min^{-1}).

S6.3 Autonomous operation

S6.3.1 Procedure for autonomous operation

[**2a** or racemic **2b**] = 1.0 mM, [(*S*)-**3** or (*R*)-**3**] = 10 mM, [DIC] = 10 mM, [MES monohydrate] = 160 mM in dioxane-d_8:D_2O (1:1 *v*/*v*) at 25 °C.

2a or racemic **2b** (1.0 µL of a 0.5 M stock solution) and hydrolysis promoter (*S*)-**3** or (*R*)-**3** (3.5 mg, 5 µmol) and MES monohydrate (17.1 mg, 80 µmol) were dissolved in dioxane-d_8/D_2O (1:1 *v*/*v*) (500 µL). DIC (5 µL of a 1.0 M stock solution in CD_3CN, 10 mM) was added and the sample was kept at 25 °C for 4 hours. The sample was acidified by the addition of 1M HCl (1 mL) and extracted with ethyl acetate (1 mL). The organic phase was dried under reduced pressure and diluted with methanol and the ratio of enantiomers was determined by chiral HPLC.

Background experiments were performed under identical conditions but lacking **2a** or racemic **2b**.

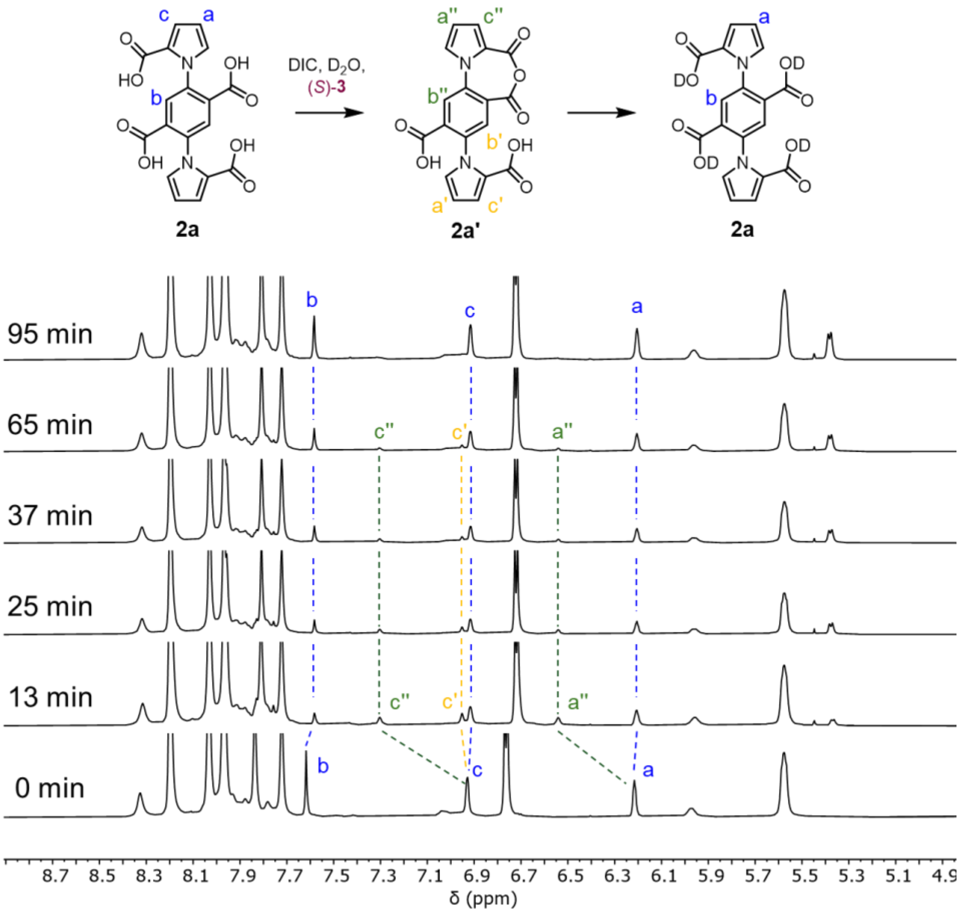

Figure S6. Autonomous operation of **2a**. Partial ^{1}H NMR spectra (dioxane-d_8:D_2O 1:1 *v*/*v*, 600 MHz, 298 K) showing the transient anhydride **2a'** formation and hydrolysis in the presence of DIC ([**2a**] = 0.5 mM, [(*S*)-**3**] = 10 mM, [DIC]$_0$ = 100 mM, [MES monohydrate] = 160 mM, pH_{obs} = 5.0).

S6.3.2 Chiral HPLC traces analysis of the autonomous operation of 2b

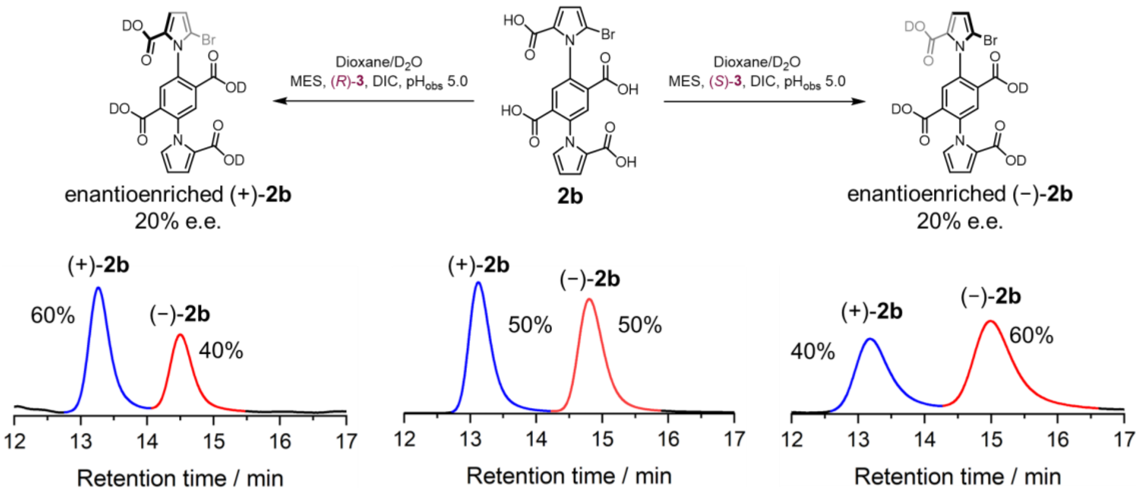

Figure S7. Chiral HPLC analysis of **2b**. Conditions: ChiralPak IA column (4.6 mm × 25 mm, 5 µm particle size), 25 °C, *i*-PrOH:*n*-hexane:0.1% TFA in CH_2Cl_2 (7:88:5 *v*/*v*/*v*), 1 mL min^{-1}. Treatment with (*R*)-**3** and DIC (left) or (*S*)-**3** and DIC (right) results in the formation of enantioenriched **2b** in 20% e.e..

S6.3.3 Monitoring autonomous operation kinetics by ^{1}H NMR

Concentration data were obtained from the relative integrals of the ^{1}H NMR signals corresponding to the CH_3 groups of DIC and DIU. The data were used to fit a line produced by a pseudo-first-order rate equation using non-linear regression. Comparison of these k_{obs} values with that of an uncatalyzed background reaction was used to assess the percentage of fuel that was used by **2a** (Figure 3) or **2b** (Figure S8) during operation.

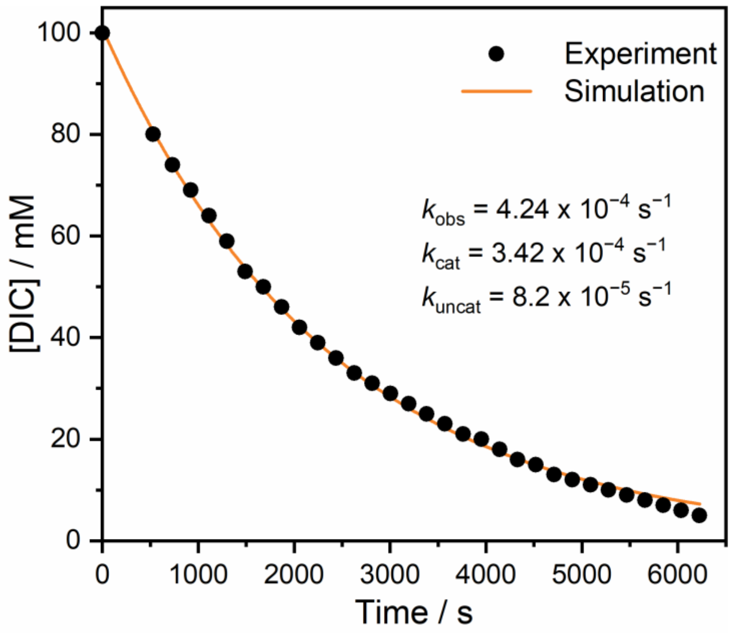

Figure S8. Kinetics of fuel consumption catalyzed by **2b** ([**2b**] = 1.0 mM, [(*S*)-**3**] = 10 mM, [DIC] = 100 mM, [MES monohydrate] = 160 mM, dioxane-d_8:D_2O (1:1 *v*/*v*) at 25 °C, pH_{obs} = 5.0). Solid lines represent the fit to pseudo-first-order kinetics (k_{obs}). Rate enhancement corresponds to 81% of fuel molecules reacting via the machine-catalyzed pathway.

S6.3.4 Calculation of the coupling efficiency η_{rot}

Directionality is only determined by the handedness of the hydrolysis promoter **3**. Under fuelling conditions, 20% e.e. for **2b** was observed and we assume the same value for motor **2a**. As the rate of interconversion between (+)-**2'** to (−)-**2'** is faster than the fuelling process, the ratio for (+)-**2a'**:(−)-**2a'** is 50%:50% when fuelling with DIC.

Following the above information, forward rotation is defined as going from (+)-**2a** to (−)-**2a** (Figure S9A). Overall, 50% of **2a** as (+)-**2a** is converted to (+)-**2a'** by consuming DIC, then 60% of those (+)-**2a'** is converted to (−)-**2a'** which is hydrolyzed to (−)-**2a** to perform the forward rotations (Forward = 0.5 × 0.6 = 0.3). The rest 40% of those (+)-**2a'** is hydrolyzed back to (+)-**2a** as a futile cycle (Figure S9C).

Then backward rotation is defined as going from (−)-**2a** to (+)-**2a** (Figure S9B). overall, 50% of **2a** as (−)-**2a** is converted to (−)-**2a'** by consuming DIC, then 60% of those (−)-**2a'** is converted to (+)-**2a'** which is hydrolyzed to (+)-**2a** to perform the backward rotations (Backward = 0.5 × 0.4 = 0.2). The rest 40% of those (−)-**2a'** is hydrolyzed back to (−)-**2a** as a futile cycle (Figure S9D).

Overall, the net rotation is equal to the forward minus backward rotations:

Forward = 0.5 × 0.6 = 0.3

Backward = 0.5 × 0.4 = 0.2

Net rotation = 0.3 − 0.2 = 0.1, coupling efficiency: η_{rot} = 10%

S6.3.5 Fuel consumption efficiency η_{fuel} and fuel efficiency $\eta_{\mathrm{rot/fuel}}$

The fuel consumption efficiency (η_{fuel}) of the motor corresponds to the amount of fuel used by the motor through the chemomecanical cycle for its forward and backward motion (Figure S9, A and B), and intrinsic futile cycles (Figure S9, C and D) correlated to the directionality of the hydrolysis promotor **3** (20% e.e.) and fuel (DIC, achiral reagent, no enantioselectivity). This value can be directly multiplied by the coupling efficiency of the motor (η_{rot}) (see section 6.3.4) to obtain the fuel efficiency ($\eta_{\mathrm{rot/fuel}}$) that corresponds to the amount of forward rotation per fuel consumed by the motor and allows us to calculate the motor rotational speed (r).

However, the catalytic efficiency (η_{cat}) is not directly equal to η_{fuel}. Indeed, some extra futile cycles (Figure S9, E and F) or side reactions (Figure S9, G) can occur leading to fuel consumption with no motions involved.

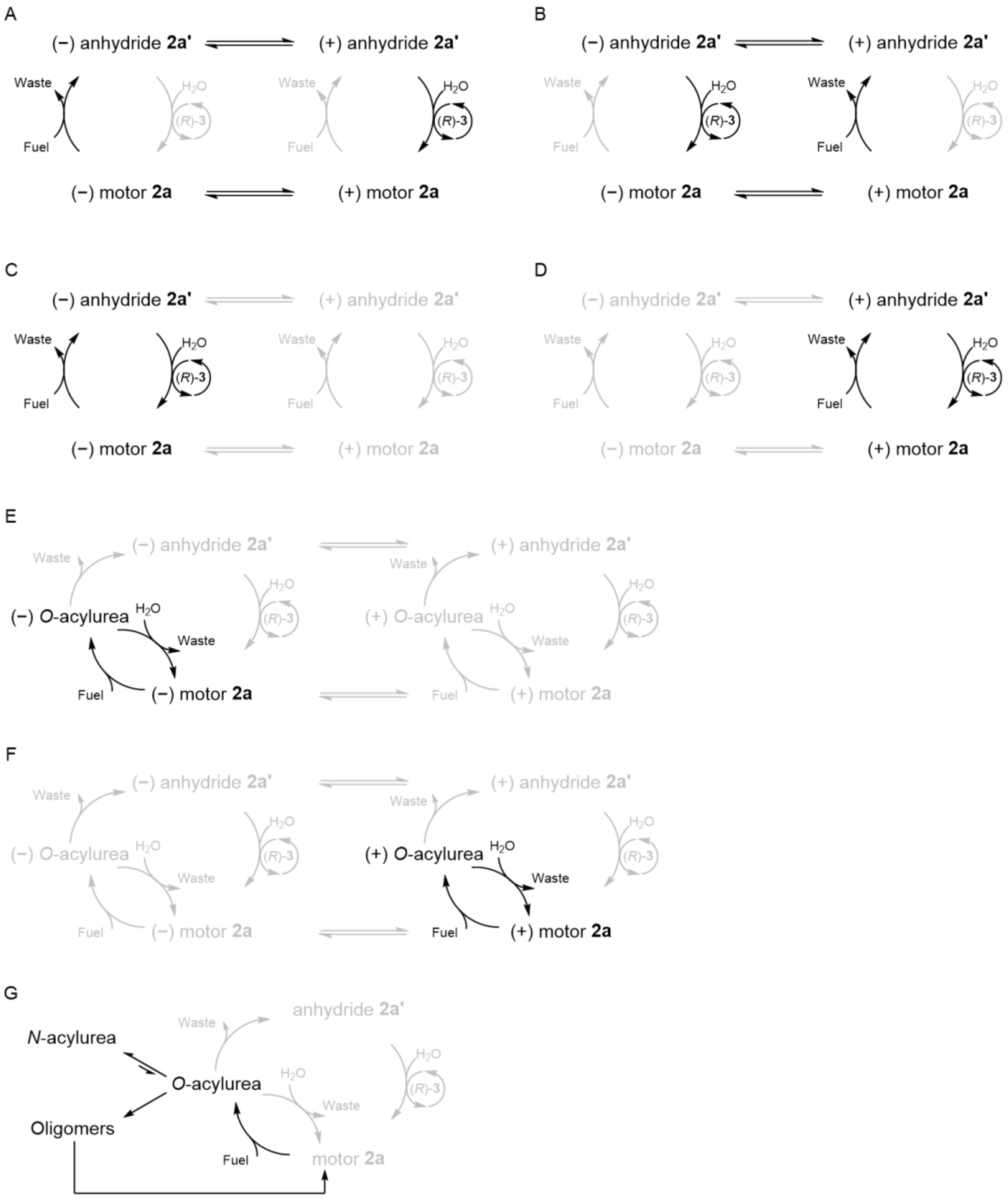

Figure S9. Couple cycles A) clockwise and B) counterclockwise, which contribute to the overall directional rotation of the motor; potential futile cycles C), D), E) and F), which consume fuel but are not involved in the net rotation; potential depletion pathways G) through the formation of *N*-acylurea or oligomers both consuming fuel. In the autonomous operation experiments, the formation of the *N*-acylurea and oligomers was not observed, which indicates the intramolecular anhydride formation is much faster than the two depletion pathways, i.e. no depletion of the motor occured during operation. As the racemization rate of **2a**, **2a'**, and **2b'** is fast relative to the fuel process, additional futile cycles were not included in this analysis.[S5]

To determine η_{fuel} we need to subtract the amount of fuel consumed by extra futile cycles or side reactions (ρ) to η_{cat}:

$$\eta_{fuel} = \eta_{cat} - \rho$$

To do so we used a statistical analysis to predict the amount of fuel that **2b** should use to reach its maximal e.e. and compare this theoretical value to the experimental one.

Statistically, maximal e.e. of **2b** is reached when all motors have reacted with at least one equivalent of DIC. In our case, to predict the amount of DIC necessary that all compound **2b** have reacted at least once with DIC we can use a Poisson distribution with a parameter λ, i.e. the equivalents of fuel. In this Poisson distribution, the probability that a single molecule **2b** uses at least one fuel is $1-e^{-\lambda}$. Considering that **2b** has a η_{cat} of 81% and a maximal e.e. of 20%, we obtain the **equation S1** for % e.e. as a function of fuel equivalent (λ).

$$\%\ e.e. = 20*(1-e^{-8.1\lambda/20}) \qquad \textbf{equation S1}$$

Therefore, plotting % e.e. of **2b** over the equivalent of fuel being consumed by the system (Figure S10) can be compared to the ideal case where no side reaction or extra futile cycles are occurring (ρ = 0).

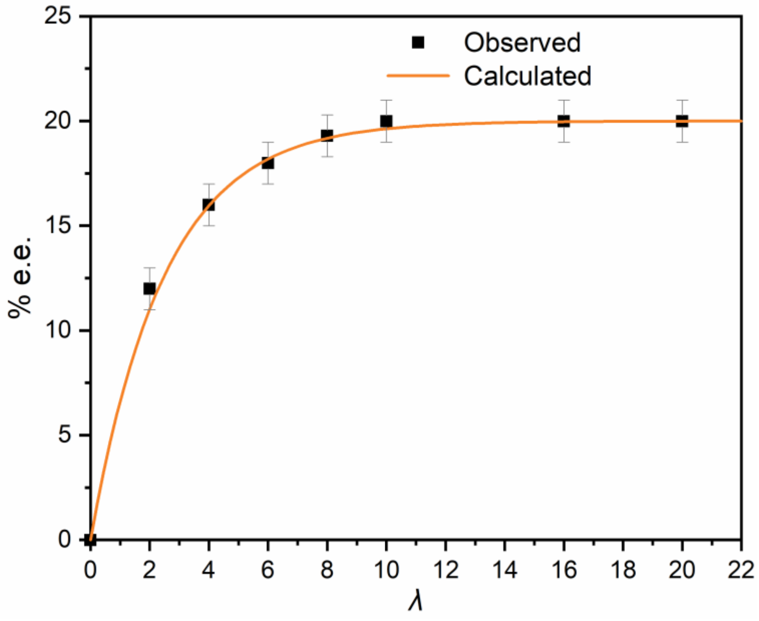

Figure S10. Directionality (% e.e.) as a function of fuel equivalent (λ) (orange curve) and experimental directionality of the motor by using different equivalents of DIC (black point). Experimental data shows the system can reach the maximum directionality by using 10 eq. fuel matching the theoretical value.

Fortunately, the statistical model fits perfectly the experimental data with an R^2 of 0.9965 indicating that **2b** and therefore motor **2a** are consuming fuels within their

chemomechanical cycle and with minimal side reactions or extra futile cycles (ρ = 0). Therefore we can consider for motor **2a** that $\eta_{\text{fuel}} = \eta_{\text{cat}}$.

Using the the probabilities for forward, backward and futile cycles, we can simulate a probability distribution function for the number of rotations in an ensemble population of motors under the chemostated experimental conditions reported in the main text Figure 4 (Figure S11).

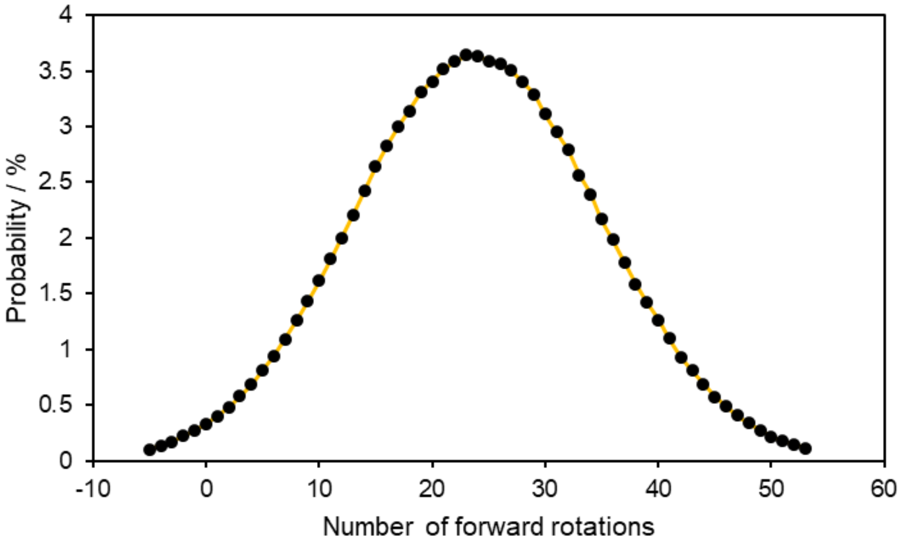

Figure S11. Probability distribution function for the rotation of motor **2a** under autonomous operation conditions [**2a**] = (5 μmol; 1 mM), under chemostated conditions by adding fuel (DIC and D_2O) at 0.8 ± 0.05 mmol h^{-1} in the presence of chiral hydrolysis promoter (*S*)-**3** (50 μmol). See main text Figure 4 for experimental data associated with this experiment.

S6.3.6 Calculation of the rotation rate

For operation of **2a** at pH$_{\text{obs}}$ 5.5: [**2a**] = 1.0 mM, [(*S*)-**3**] = 10 mM, [DIC] = 100 mM, [MES buffer] = 100 mM (pH$_{\text{obs}}$ 5.10 for 1 M stock solution in D_2O), dioxane-*d*$_8$/D_2O (7:3 *v*/*v*) at room temperature.

For operation of **2a** at pH$_{\text{obs}}$ 5.0: [**2a**] = 1.0 mM, [(*S*)-**3**] = 10 mM, [DIC] = 100 mM, [MES monohydrate] = 160 mM, dioxane-*d*$_8$/D_2O (1:1 *v*/*v*) at room temperature.

For operation of **1** at pH$_{\text{obs}}$ 5.0: [**1**] = 1.0 mM, [(*S*)-**3**] = 10 mM, [DIC] = 100 mM, [MES monohydrate] = 160 mM, dioxane-*d*$_8$/D_2O (1:1 *v*/*v*) at room temperature.

2a or **1** (0.5 μL of a 1.0 M stock solution) and hydrolysis promoter (*S*)-**3** (3.5 mg, 5 μmol) was dissolved in MES monohydrate or MES-buffered dioxane-d_8/D_2O mixture (0.5 mL). DIC (6.3 mg, 50 μmol) was added and the reaction was monitored by ^{1}H NMR spectroscopy. Background experiments were performed under identical conditions but lacking **2a** or **1**. Concentration data were obtained from the relative integrals of the ^{1}H NMR signals corresponding to the CH_3 groups of DIC and DIU.

Pseudo-first-order kinetics provided an excellent fit for DIC hydrolysis catalyzed by **2a** (Figure 3 and Figure S12). The concentration of DIC over time can be expressed by **equation S2** (rate constant k_{obs} was shown as k). The derivation of **equation S2** gives **equation S3**, which represents the consumption rate of DIC per second (Figure S12A). Knowing the coupling efficiency of the motor η_{rot} (see section S6.3.4) and the fuel consumption efficiency η_{fuel} (see section S6.3.5), we can determine the rotational fuel efficiency $\eta_{rot/fuel}$ in **equation S4**. Then applying the $\eta_{rot/fuel}$ from **equation S3** to **equation S4** gives motor speed with **equation S5**. Finally, integrating **equation S5** gives the total number of net rotations performed by motor **2a** as **equation S6**.

[DIC] as a function of time t (mM): $[DIC]_t = [DIC]_0 e^{-kt}$ **equation S2**

[DIC] consuming rate (mM·s^{-1}): $[DIC]_t{}' = -k[DIC]_0 e^{-kt}$ **equation S3**

fuel efficiency (rotations·mM^{-1}): $\eta_{rot/fuel} = \eta_{rot} \times \eta_{fuel}$ **equation S4**

motor speed (rotations·s^{-1}): $r = \left| [DIC]' \times \eta_{rot/fuel} \right| = \eta_{rot/fuel}\, k[DIC]_0 e^{-kt}$ **equation S5**

total net rotations of the motor (rotations)

$$\int_{t_0}^{t} r = -\eta_{rot/fuel}[DIC]_0 e^{-kt} + \eta_{rot/fuel}[DIC]_0$$ **equation S6**

At pH_{obs} 5.0, the model indicates that each motor would achieve 9 rotations on average at a maximum rate ($r_{initial}$) of about 0.43 rotations per minute (or 1 rotation every 2.3 minutes). The motor would use 90% of the available fuel to achieve this, with the other 10% being used by the background processes, thereby achieving approximately 1 net rotation per 11 units of fuel consumed by the system.

At pH_{obs} 5.5, the model indicates that each motor would achieve 9 rotations on average at a maximum rate ($r_{initial}$) of about 0.14 rotations per minute (or 1 rotation every 7 minutes). The motor would use 99% of the available fuel to achieve this, with the other 1% being used by the background process, thereby achieving approximately 1 net rotation per 10 units of fuel consumed by the system.

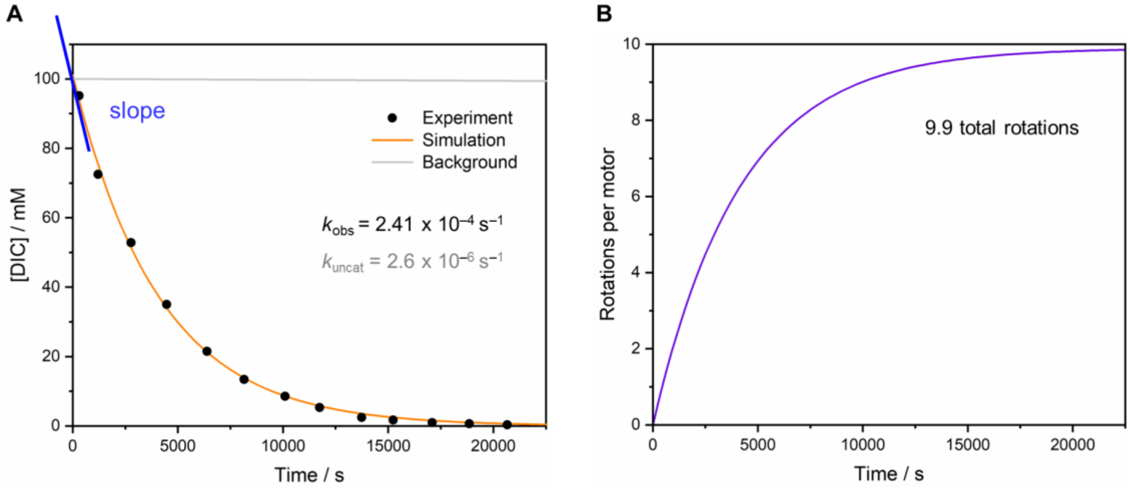

Figure S12. A) Kinetics of carbodiimide hydration at pH_{obs} 5.5 in the absence and presence of **2a** (1.0 mM), determined by ^{1}H NMR spectroscopy. Solid lines represent the fit to pseudo-first-order kinetics. Rate enhancement corresponds to 99% of fuel molecules reacting via the motor-catalyzed pathway. The slope (blue) of the fuel consumption curve (orange) shows the [DIC] consumption rate. B) The cumulative count of rotations per motor achieved during this operation, according to the simulation.

S6.3.7 Operation of single motor 1

At pH_{obs} = 5.0, single motor **1** exhibits a catalytic efficiency (57%) and performs a directional rotation of approximately 0.06 rpm at 100 mM fuel, i.e., a directional rotation every 17 minutes. In contrast, the dual motor shows a catalytic efficiency of 90% and achieves a rotation speed of 0.43 rpm at the same fuel concentration, making it 7 times faster than the single motor. The higher catalytic efficiency and negligible observation of *N*-acylurea formation when operating dual motor **2** can be attributed to the more nucleophilic feature of its structure.

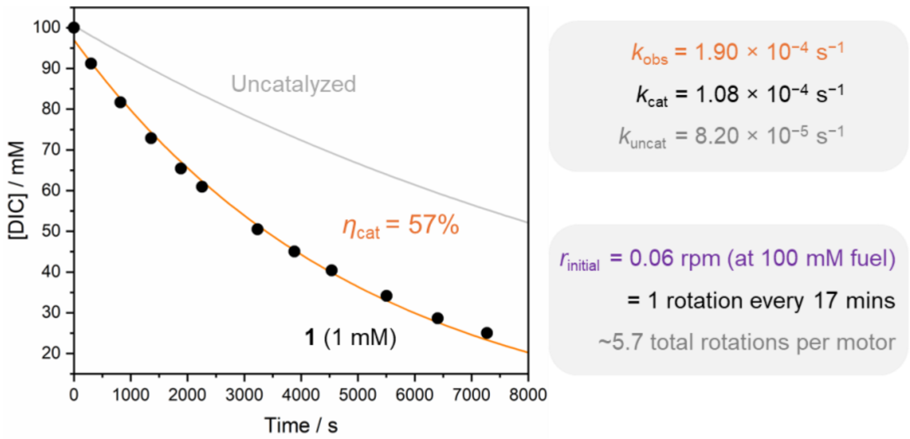

Figure S13. Kinetics of DIC hydration in the absence and presence of **1** (1.0 mM) at pH_{obs} 5.0, determined by ^1H NMR spectroscopy. Solid lines (orange and grey) represent the fit to pseudo-first-order kinetics (k_{obs}).

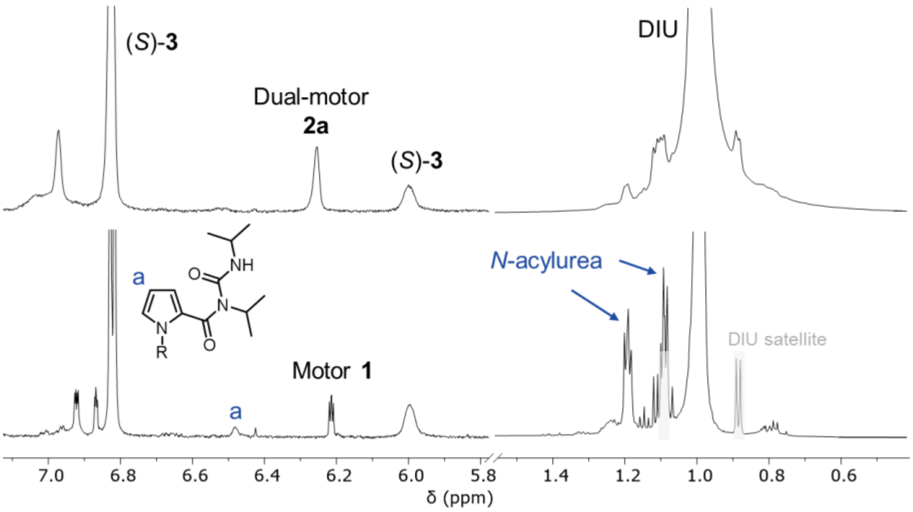

Figure S14. Partial ^1H NMR (dioxane-d_8/D_2O 1:1 *v*/*v*, 600 MHz, 298 K) of operation of motor **2a** at pH_{obs} 5.0 after fueling with 100 Mm DIC (top), operation of motor **1** at pH_{obs} 5.0 after fueling with 100 mM DIC (bottom). Motor **1** showed a 30% depletion due to *N*-acylurea formation, while dual motor **2a** displayed negligible depletion. The region of 5.8–7.1 ppm is scaled vertically 10× compared to the region of 0.5–1.5 ppm.

We used the experimental data from the previous study[S6] and applied the methodology described above to calculate the initial rotation speed of single motor **1**. The results show that motor **1** operates at a speed of 0.318 rotations per hour (0.31 rotation per hour reported previously). This result supports that both methodologies give the same results.

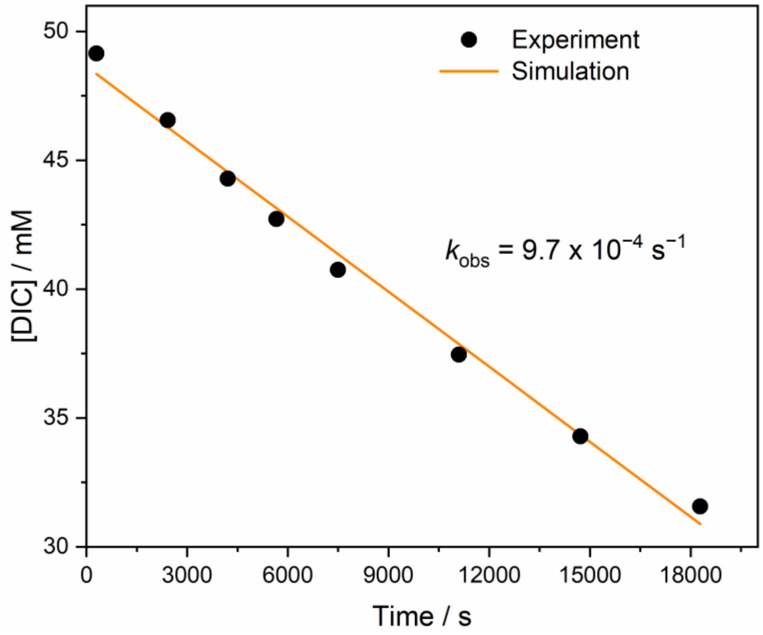

Figure S15. Kinetics of DIC hydration in the presence of **1**, determined by ^{1}H NMR spectroscopy.

As reported in the previous paper, the initial fuel consumption of the DIC showed a linear relationship over time, thus

[DIC] as a function of time t (mM): $[DIC] = -kt + [DIC]_0 = -9.7\times10^{-4}\ t + 50$

[DIC] consuming rate ($mM\cdot s^{-1}$): $[DIC]_t' = -k = -9.7\times10^{-4}$

fuel efficiency ($rotations\cdot mM^{-1}$): $\eta_{rot/fuel} = \eta_{rot} \times \eta_{fuel} = 0.91\times0.1 = 0.091$

motor speed ($rotations\cdot s^{-1}$): $r = \mid [DIC]_t' \times \eta_{rot/fuel} \mid = 9.7\times10^{-4}\times0.091 = 8.827\times10^{-5}$ (i.e. 0.318 rotation per hour)

S6.4 Consistency of pH during fuelling

While pH 5.0 is outside of the buffer range of MES, we determined that the pH of the system (SevenCompact S220-Basic, pH/Ion benchtop meter) remained constant during the reaction, thus allowing kinetic measurements to be made with confidence.

Table S1. Reaction pH_{obs} as a function of time for **2a** (1 mM) under autonomous operation conditions.

Reaction time (min)	pH_{obs}
0	4.92
5	4.94
10	4.96
15	4.99
20	5.00
40	5.00
60	5.00
120	4.97

Table S2. Reaction pH_{obs} as a function of time for **2b** (1 mM) under autonomous operation conditions.

Reaction time (min)	pH_{obs}
0	4.91
5	4.94
10	4.97
15	5.00
20	5.00
40	5.01
60	5.00
120	4.98

S7. Operation of 2a under chemostated conditions

[**2a**] = 1.0 mM, [(*S*)-**3** or (*R*)-**3**] = 10 mM, [DIC] = 310 mM, [MES monohydrate] = 160 mM in dioxane:D_2O (1:1 *v*/*v*) at 25 °C.

2a (1.9 mg, 5 μmol), chiral hydrolysis promoter (*S*)-**3** or (*R*)-**3** (35 mg, 50 μmol) and MES monohydrate (171 mg, 800 μmol), DIC (32 mg, 250 μmol) were dissolved in a mixture of dioxane-d_8/D_2O (1:1 *v*/*v*) (5 mL), and the solution was stirred at 25 °C. DIC (168 mg, 1.3 mmol) and D_2O (27 mg, 1.3 mmol) were added by syringe pump at 0.8 ± 0.05 $mmol \cdot h^{-1}$. Samples (50 μL) were periodically removed and analyzed by ^{1}H NMR spectra.

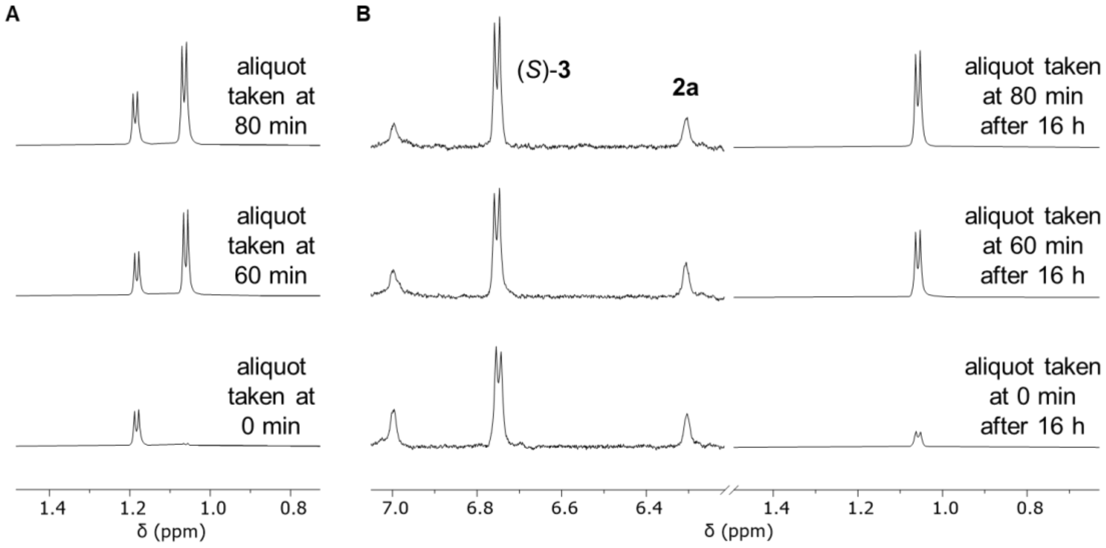

Figure S16. Partial ^{1}H NMR (600 MHz, CD_3CN, 298 K) of A) the aliquots taken at different time points and measured after 5 min to monitor the concentration of DIC, and B) after 16 h (until DIC consumed completely) to monitor the concentration of motor **2a**, which indicates almost no depletion of motor **2a**. The region of 6.2–7.0 ppm is scaled vertically 400× compared to the region of 0.8–1.4 ppm.

S8. Operation of dual motor 2 under conditions optimized for high directionality

Using recently reported operational conditions for fueling motor **1** with high directionality,[S7] we performed the operation for compound **2b** under similar conditions, achieving a directionality of 11.5:1.

In a round bottom flask were added 1.0 mL of dioxane, 0.8 mL D_2O, and 0.2 mL (100 mM) of MES buffer solution (1.0 M in D_2O). To the stirred mixture, 4.0 mM of (*S*)-**4** was added along with 1.0 mM of compound **2b**. A syringe pump was loaded with a 0.24 M solution of fuel (*S,S*)-*N,N*-di(isopropylbenzyl)carbodiimide in dioxane and injected into the reaction mixture at a rate of 25 μL/h for ~4 hours. The ratio of

enantiomers was determined by chiral HPLC. (ChiralPak IA column (4.6 mm × 25 mm, 5 µm particle size), 25 °C, *i*-PrOH:*n*-hexane:0.1% TFA in CH_2Cl_2 (7:88:5 *v*/*v*/*v*), 1 mL min^{-1}).

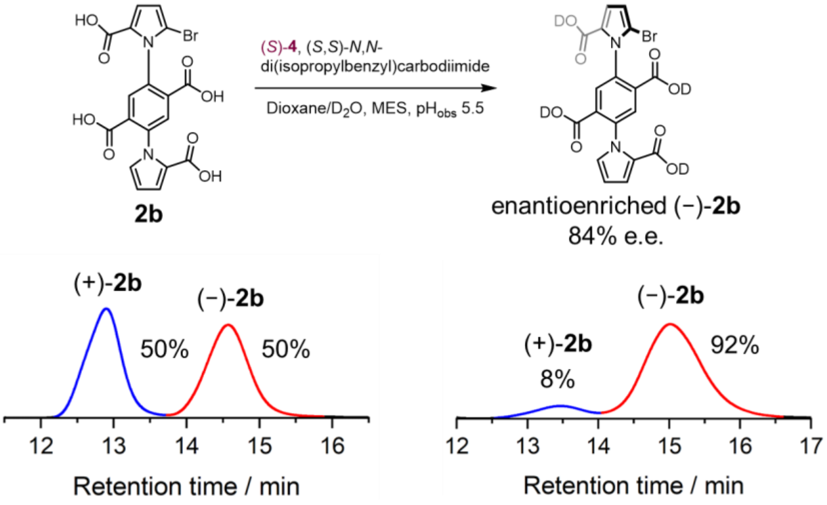

Figure S17. Chiral HPLC analysis of **2b**. Treatment with (*S*)-**4** and (*S,S*)-*N,N*-di(isopropylbenzyl)carbodiimide results in the formation of enantioenriched (–)-**2b** in 84% e.e..

S9. Crystallographic data

S9.1 SCXRD experimental details

Data collection: X-ray diffraction data for compounds **2a** and **2b** were collected using a dual wavelength Rigaku FR-X rotating anode diffractometer using CuKα (λ = 1.54146 Å) radiation, equipped with an AFC-11 4-circle quarter-χ goniometer, VariMAX™ microfocus optics, a Hypix-6000HE detector and an Oxford Cryosystems 800 plus nitrogen flow gas system, at a temperature of 100K. Data were collected and reduced using CrysAlisPro v43.[S8] Absorption correction was performed using empirical methods (SCALE3 ABSPACK) based upon symmetry-equivalent reflections combined with measurements at different azimuthal angles.

S9.2 Crystal structure determination and refinements

The crystal structure was solved and refined against all F^2 values using the SHELX and Olex2 suite of programs.[S9,S10] Coordinates for all non-hydrogen atoms were freely refined and atomic displacement parameters were freely refined anisotropically. Hydrogen atoms were constrained to idealised positions with the coordinates refined to ride with the parent atom. Hydrogen isotropic atomic displacement parameters were constrained to ride with the parent atom with an appropriate multiplier for the

hybridisation. Both molecules crystallise about the crystallographic inversion centre and are modelled with half a molecule in the asymmetric unit. The structure of **2b** is disordered about the crystallographic inversion centre, with Br1 and H5 being refined in separate parts with half occupancy. The carboxylic acid labelled C9 O3 O4 H3 was also found to be disordered and was modelled over two positions in separate parts. The occupancies for the disordered parts were freely refined competitively against a single free variable, such that the overall occupancy of the disordered component equals 1, giving a relative occupancy of 0.736(7):0.264(7). Solvent molecules in **2b** were found to be highly disordered and were modelled using a solvent mask to account for the intensity contributions of the solvent void. The electron count for the modelled solvent void is 56 electrons for the whole unit cell, corresponding to two methanol and two water molecules.

CCDC 2407393-2407394 contain the supplementary crystallographic data for this paper. These data can be obtained free of charge via https://www.ccdc.cam.ac.uk/structures/, or by emailing data_request@ccdc.cam.ac.uk, or by contacting The Cambridge Crystallographic Data Centre, 12 Union Road, Cambridge, CB2 1EZ, UK; fax: +44 1223 336033.

Table S3. Crystallographic details

Identification code	**2a**	**2b**
Empirical formula	$C_{24}H_{26}N_4O_{10}$	$C_{20}H_{23}BrN_2O_{12}$
Formula weight	530.49	563.31
Temperature/K	99.97(11)	99.97(14)
Crystal system	triclinic	triclinic
Space group	P-1	P-1
a/Å	7.1738(6)	6.8519(3)
b/Å	8.6805(6)	8.9968(4)
c/Å	10.6479(7)	11.6781(5)
α/°	83.817(6)	97.927(3)
β/°	77.387(6)	99.145(4)
γ/°	79.732(6)	102.776(4)
Volume/Å^3	635.07(9)	681.86(5)
Z	1	1
ρ_{calc}g/cm^3	1.387	1.372
μ/mm^{-1}	0.930	2.582
F(000)	278.0	288.0
Crystal size/mm^3	0.116 × 0.072 × 0.022	0.08 × 0.07 × 0.06
Radiation	Cu Kα (λ = 1.54184)	Cu Kα (λ = 1.54184)
2Θ range for data collection/°	8.532 to 152.184	10.248 to 152.764
Index ranges	-8 ≤ h ≤ 9, -10 ≤ k ≤ 10, -13 ≤ l ≤ 13	-8 ≤ h ≤ 8, -11 ≤ k ≤ 11, -13 ≤ l ≤ 14
Reflections collected	7180	7993
Independent reflections	2556 [R_{int} = 0.0239, R_{sigma} = 0.0293]	2768 [R_{int} = 0.0291, R_{sigma} = 0.0316]
Data/restraints/parameters	2556/0/182	2768/90/165
Goodness-of-fit on F^2	1.079	1.109
Final R indexes [I>=2σ (I)]	R_1 = 0.0382, wR_2 = 0.0997	R_1 = 0.0588, wR_2 = 0.1846
Final R indexes [all data]	R_1 = 0.0420, wR_2 = 0.1020	R_1 = 0.0622, wR_2 = 0.1874
Largest diff. peak/hole / e Å^{-3}	0.52/-0.20	0.28/-0.38

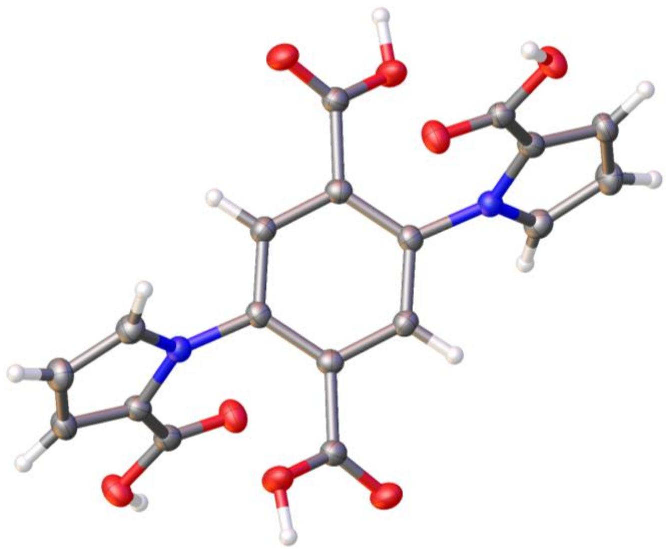

Figure S18. Structure of **2a** in the crystal. Solvent DMF omitted for clarity. C = grey, N = blue, O = red, H = white.

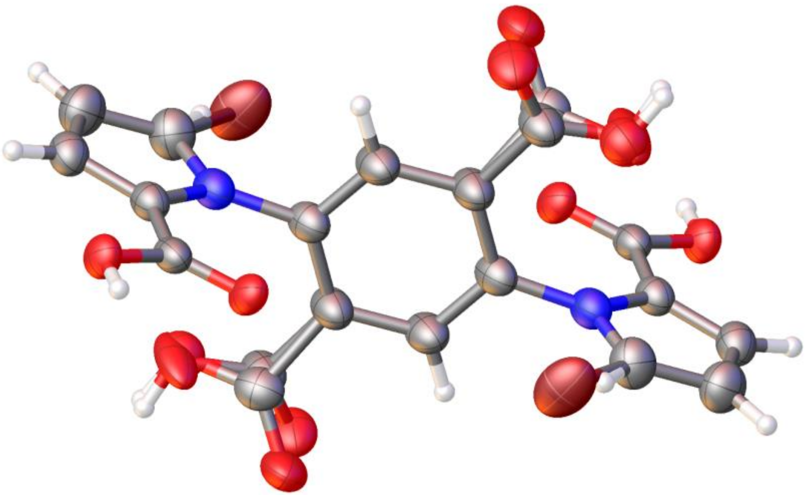

Figure S19. Structure of **2b** in the crystal. C = grey, N = blue, O = red, Br = maroon, H = white.

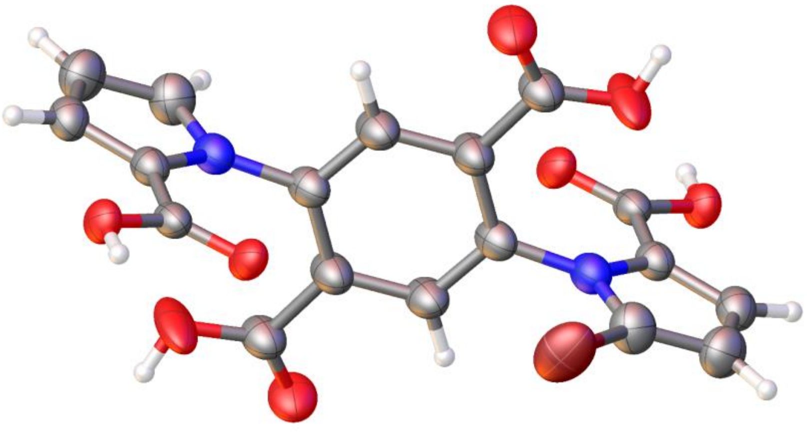

Figure S20. Structure of **2b** with disorder removed for clarity. A major occupancy component for the disordered carboxylic acid was selected. C = grey, N = blue, O = red, Br = maroon, H = white.

S10. NMR spectra

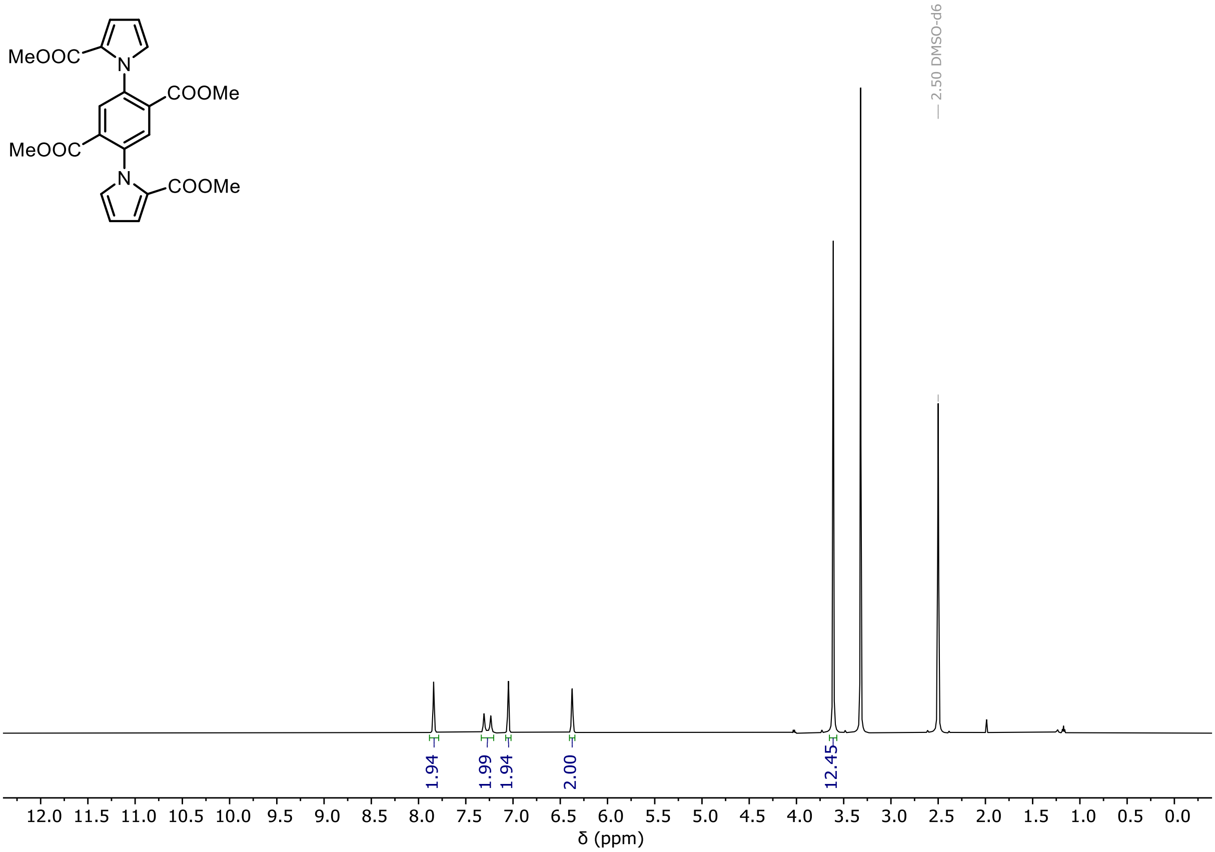

Spectrum S1: ^{1}H NMR spectrum (600 MHz, $(CD_3)_2SO$) of **S3**.

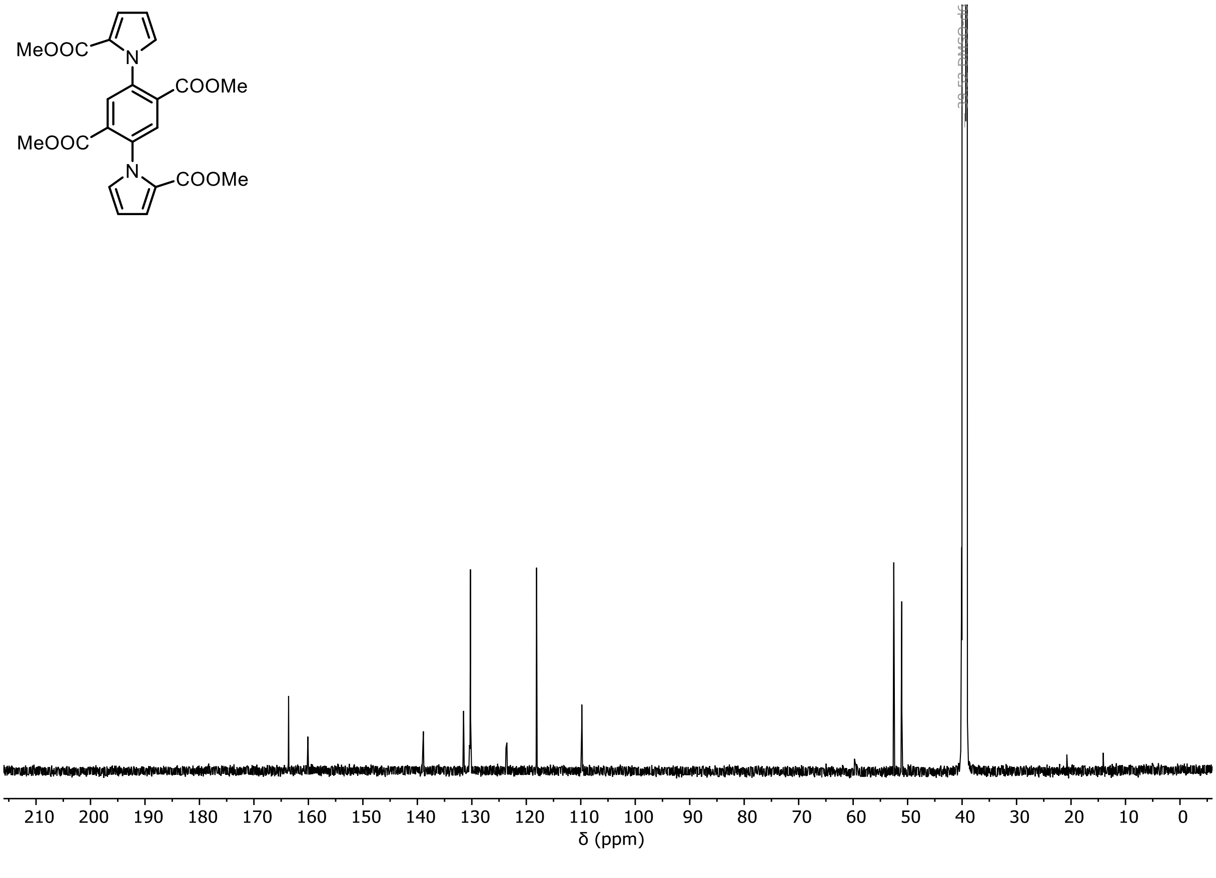

Spectrum S2: ^{13}C NMR spectrum (151 MHz, $(CD_3)_2SO$) of **S3**.

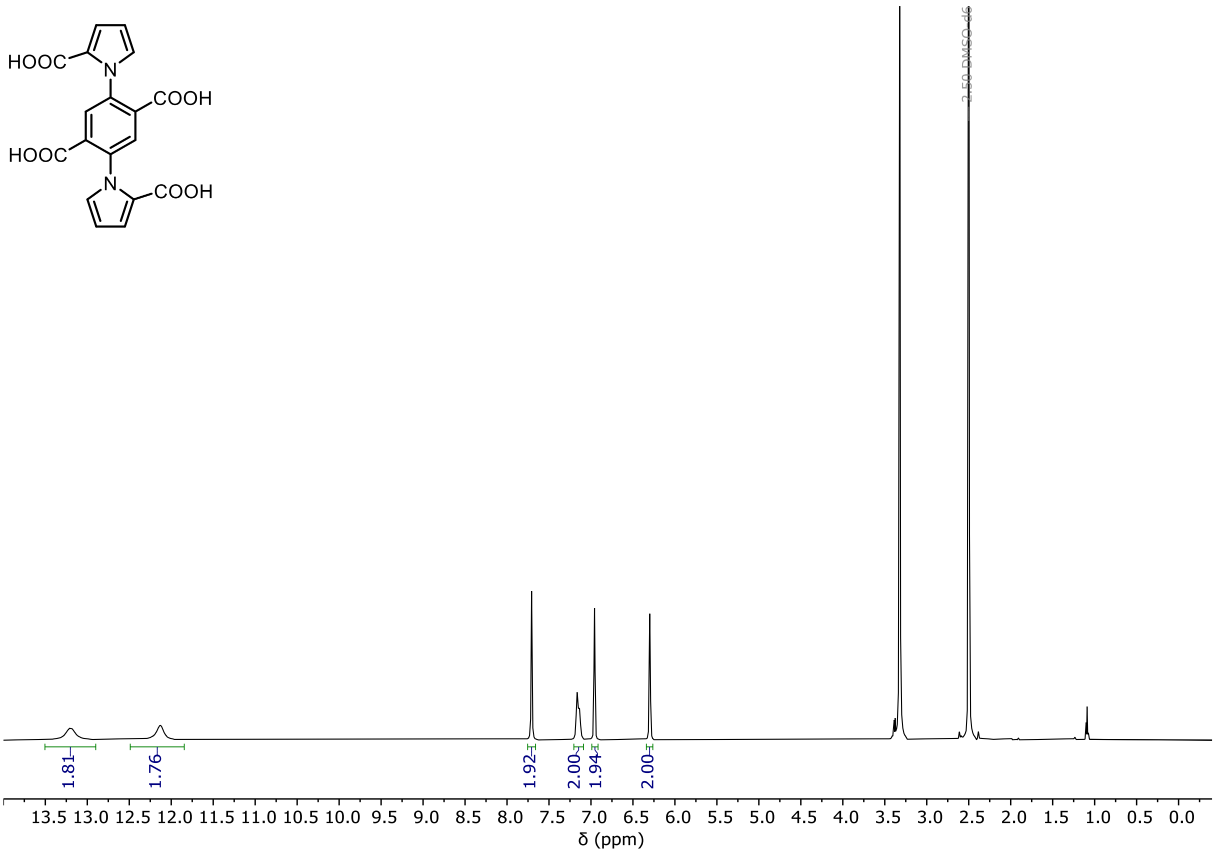

Spectrum S3: ^{1}H NMR spectrum (600 MHz, $(CD_3)_2SO$) of **2a**.

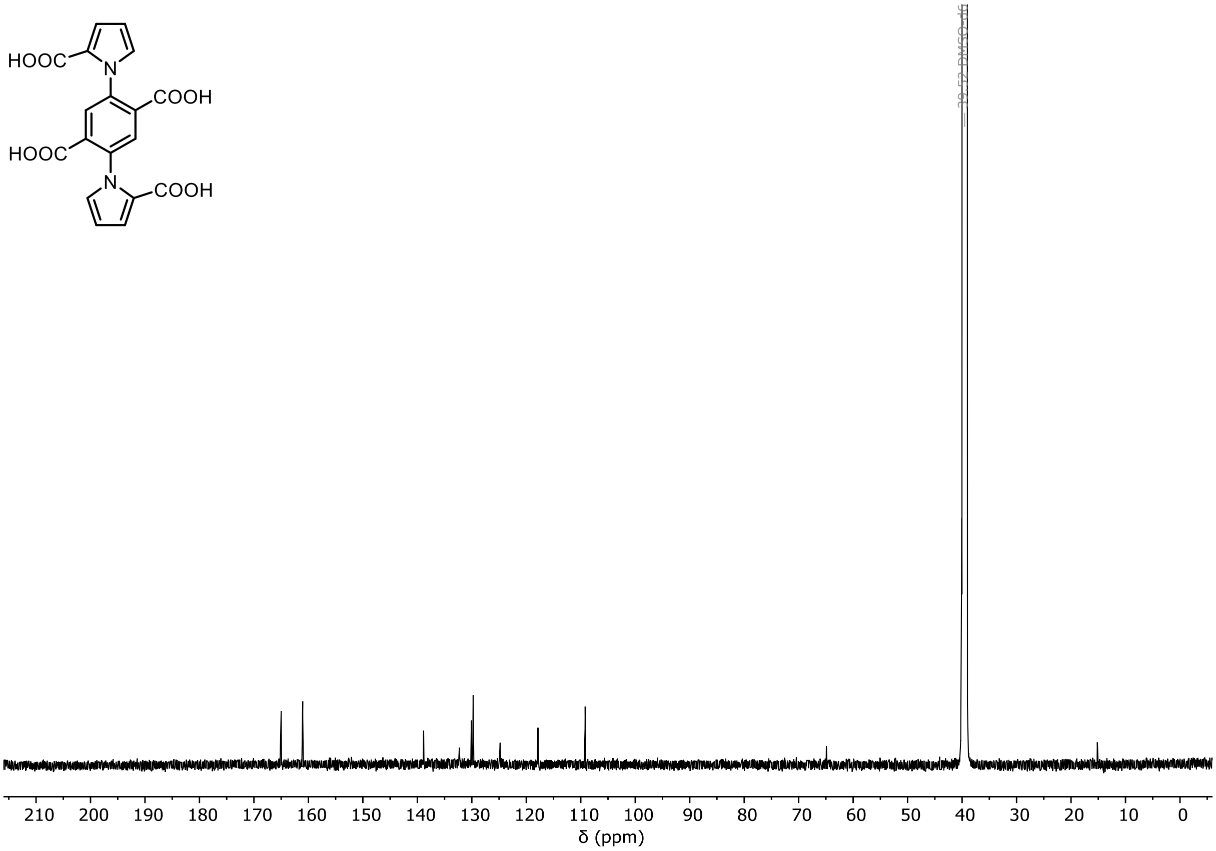

Spectrum S4: ^{13}C NMR spectrum (151 MHz, $(CD_3)_2SO$) of **2a**.

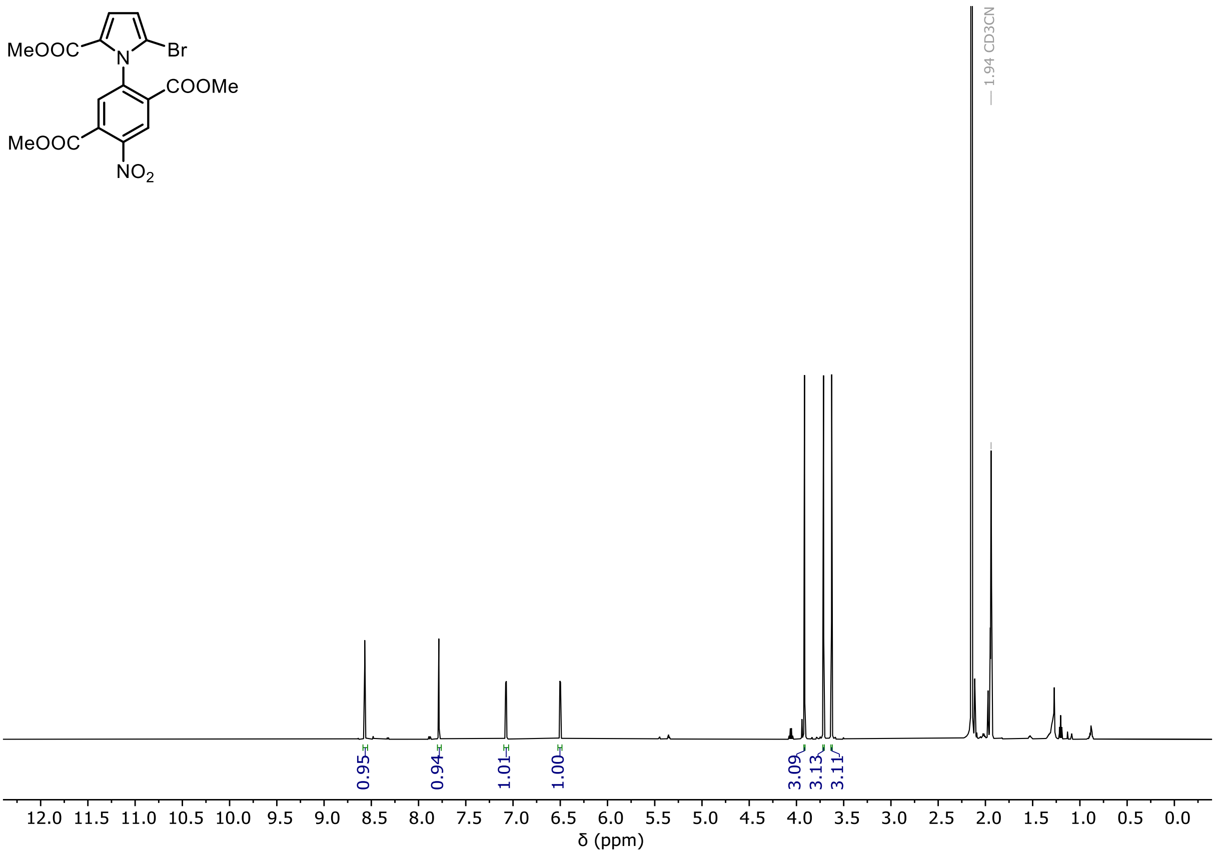

Spectrum S5: ^{1}H NMR spectrum (600 MHz, CD_3CN) of **S6**.

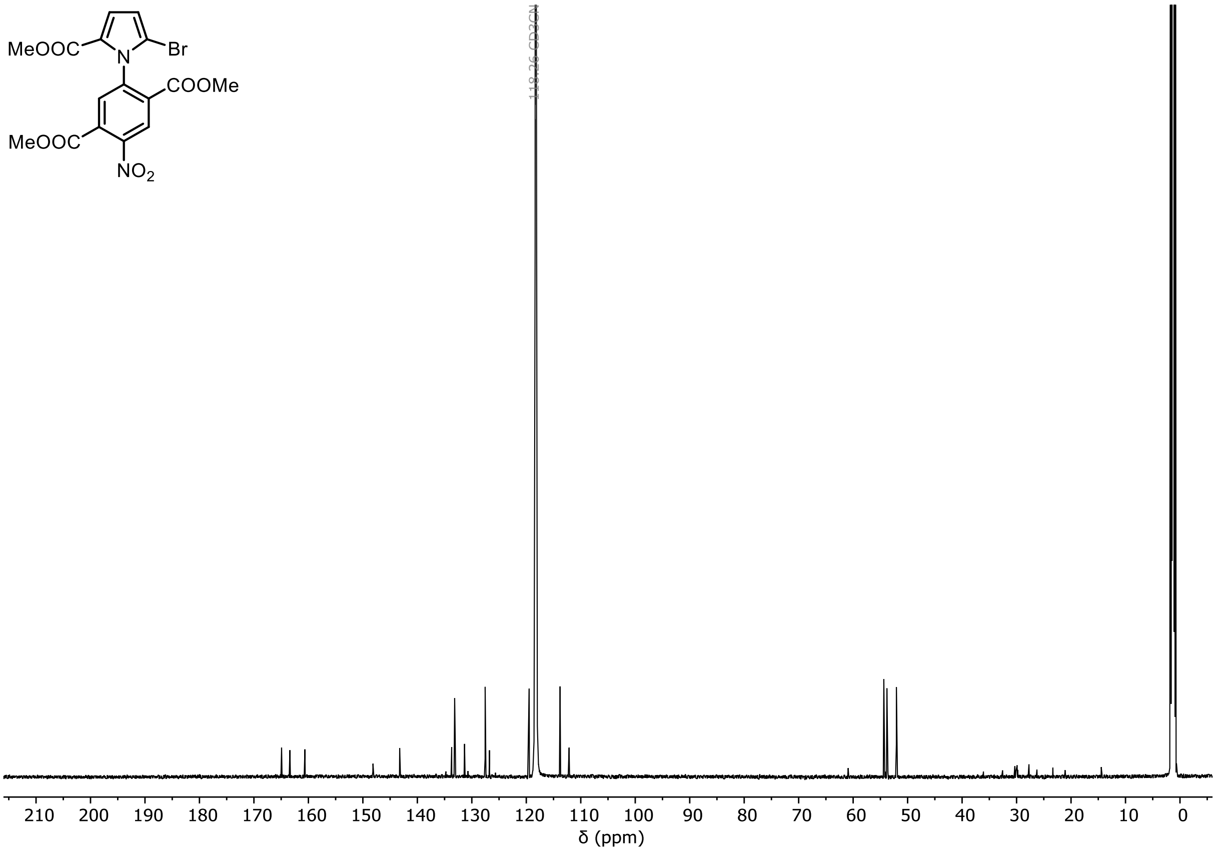

Spectrum S6: ^{13}C NMR spectrum (151 MHz, CD_3CN) of **S6**.

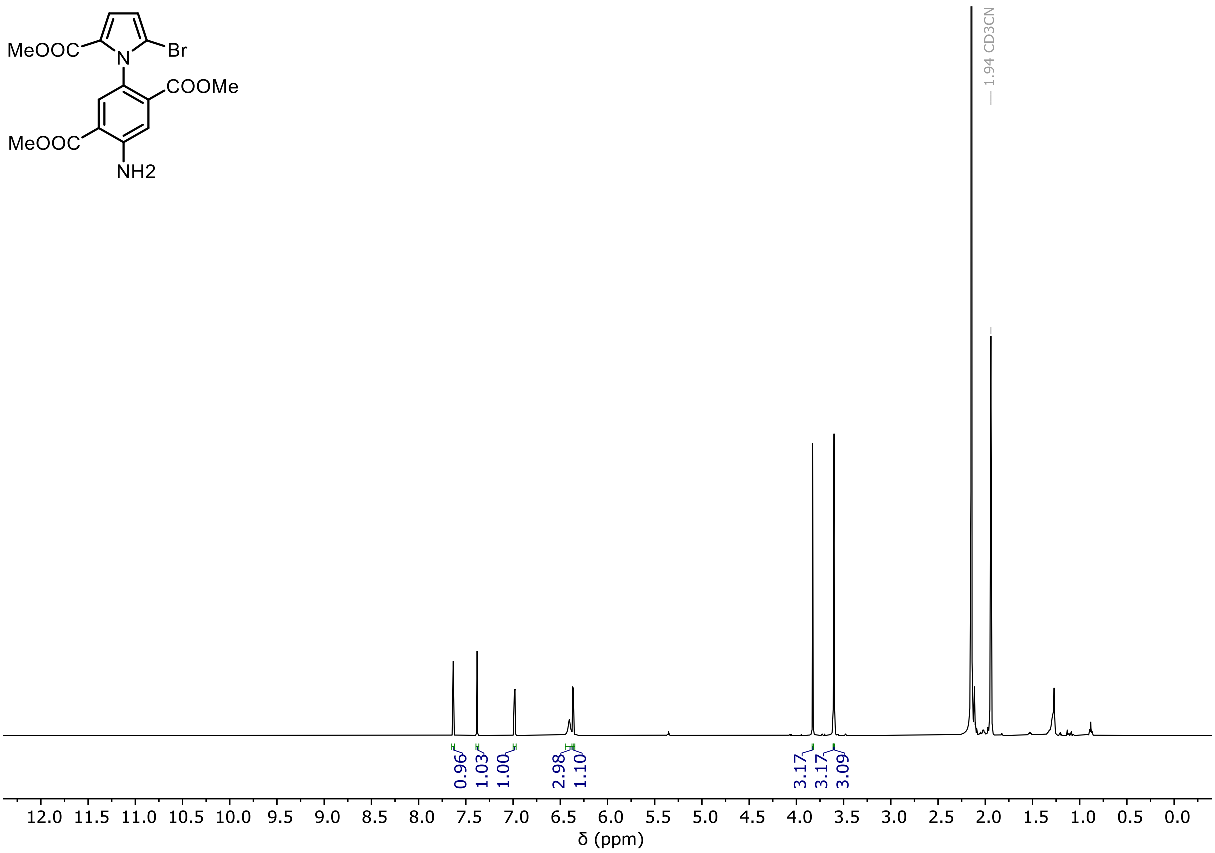

Spectrum S7: ^{1}H NMR spectrum (600 MHz, CD_3CN) of **S7**.

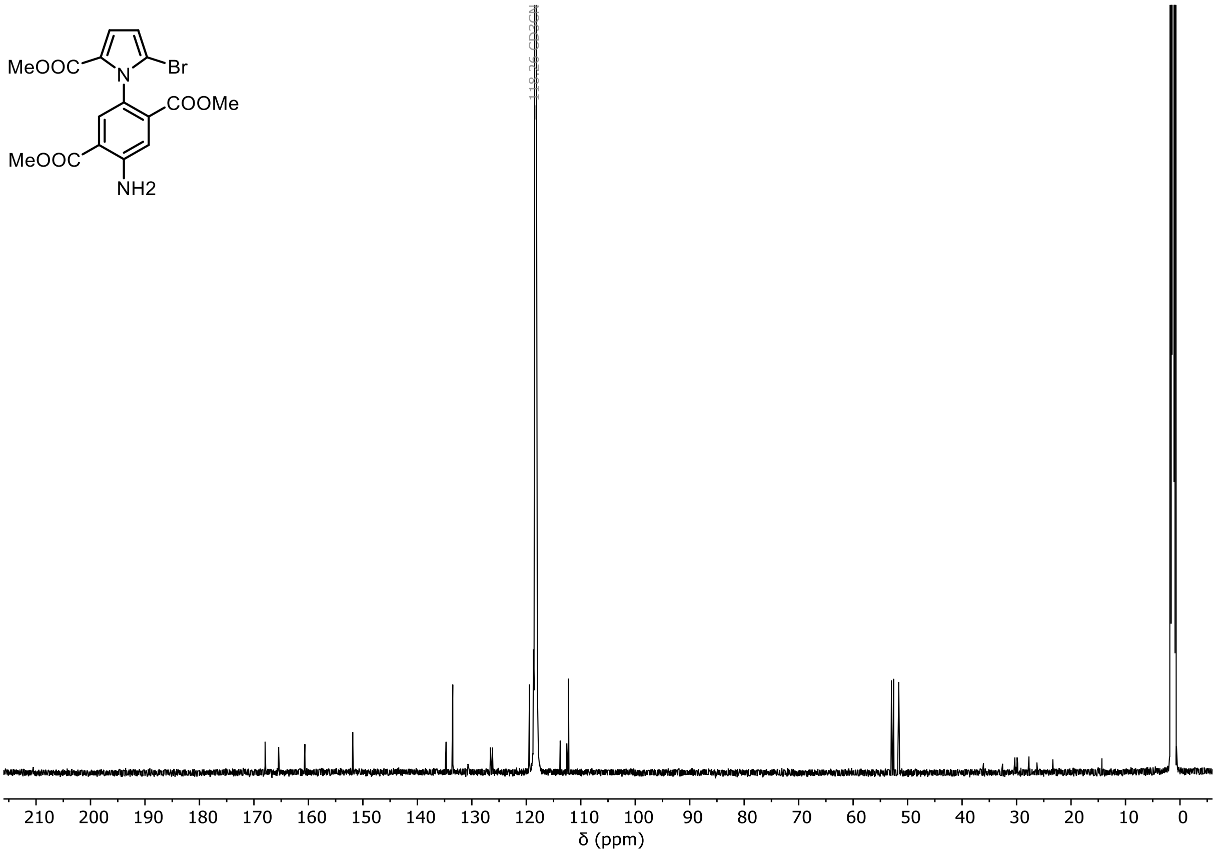

Spectrum S8: ^{13}C NMR spectrum (151 MHz, CD_3CN) of **S7**.

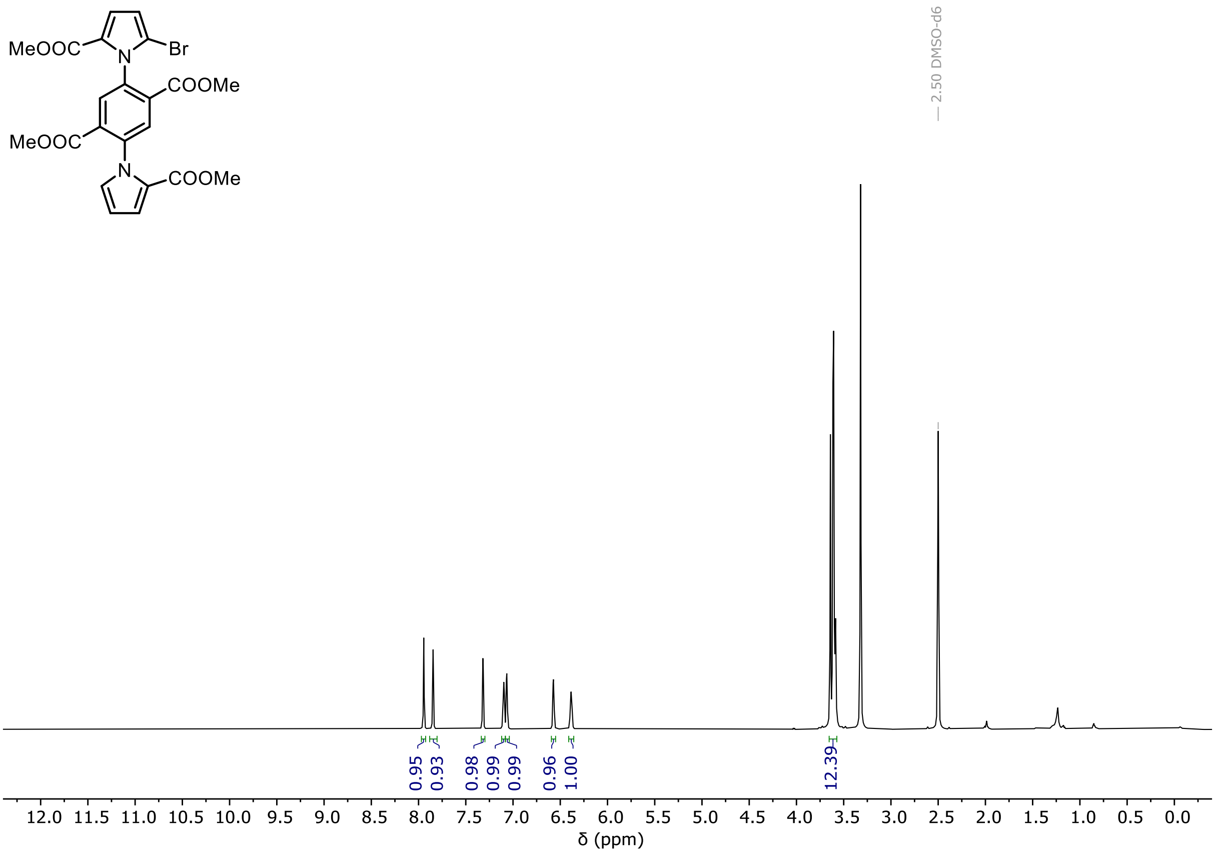

Spectrum S9: ^{1}H NMR spectrum (600 MHz, $(CD_3)_2SO$) of **S8**.

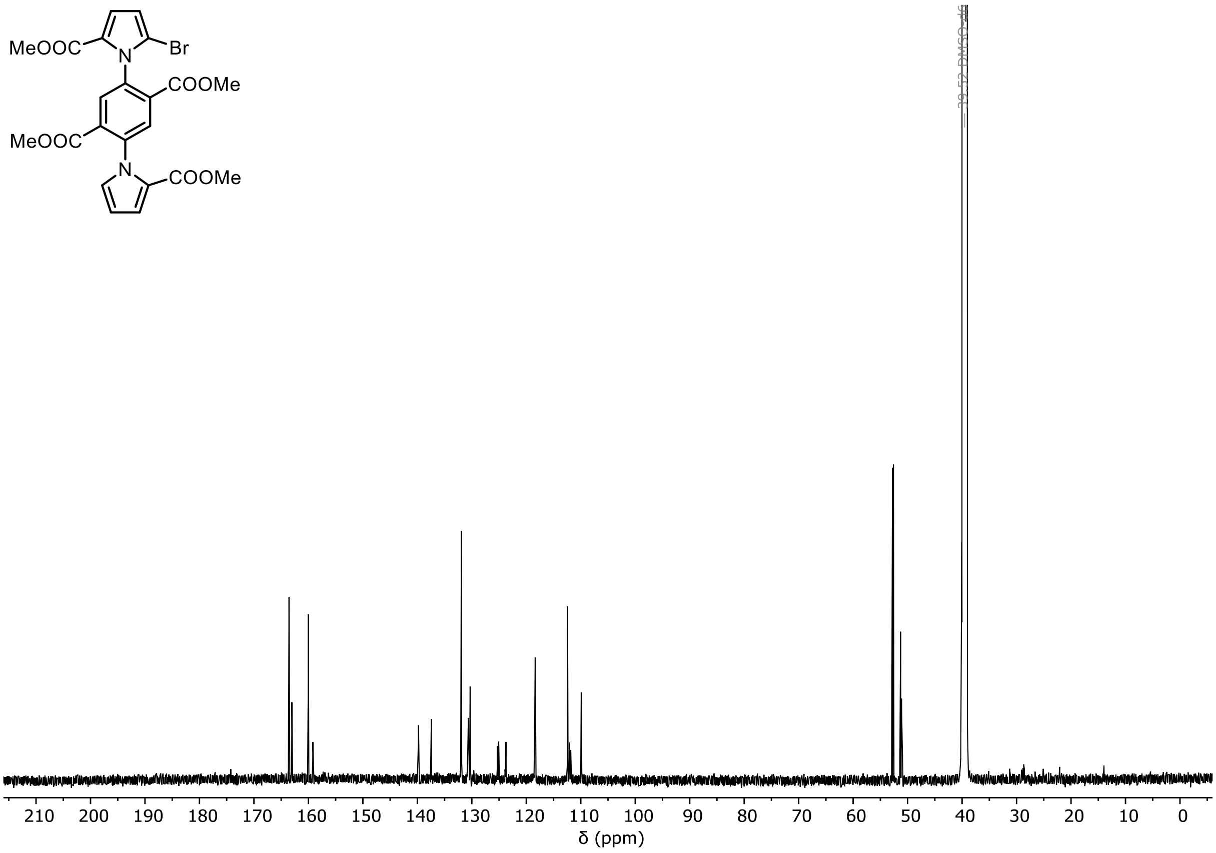

Spectrum S10: ^{13}C NMR spectrum (151 MHz, $(CD_3)_2SO$) of **S8**.

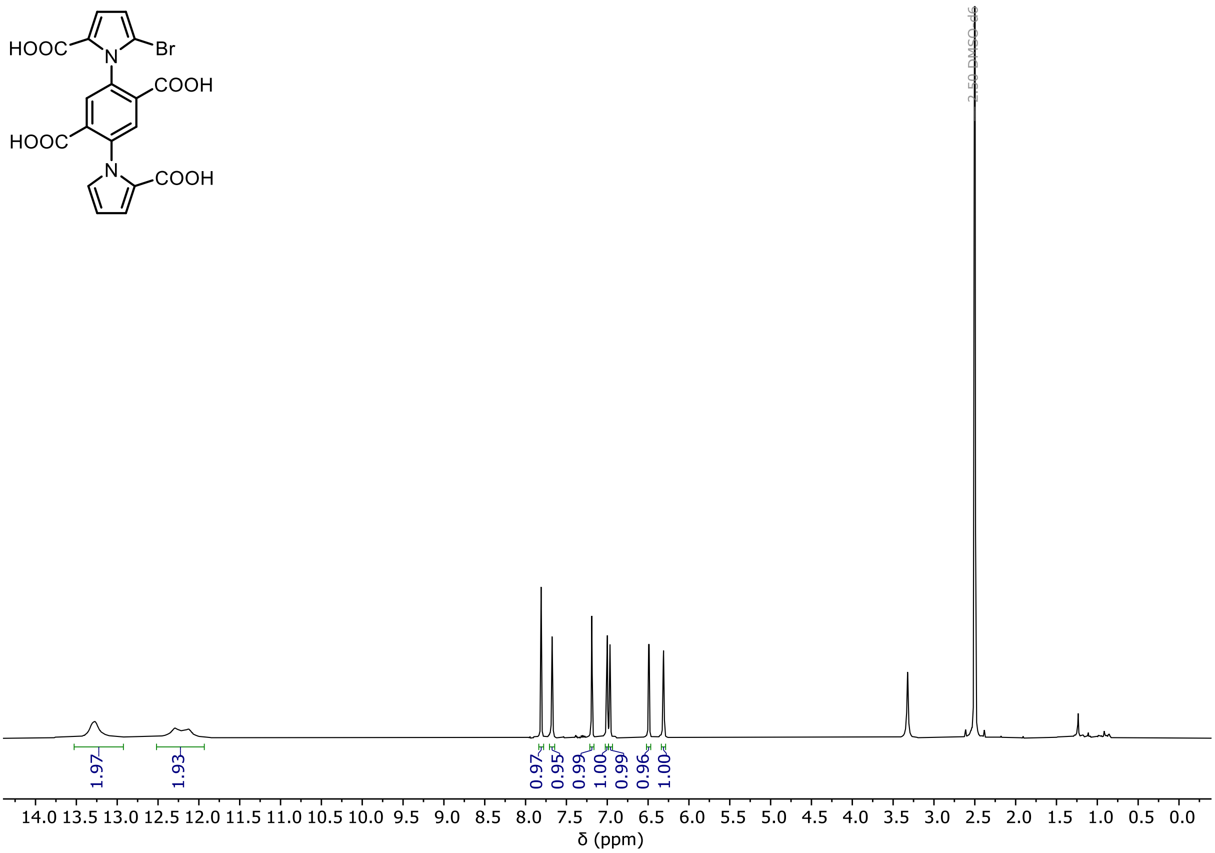

Spectrum S11: ^{1}H NMR spectrum (600 MHz, $(CD_3)_2SO$) of **2b**.

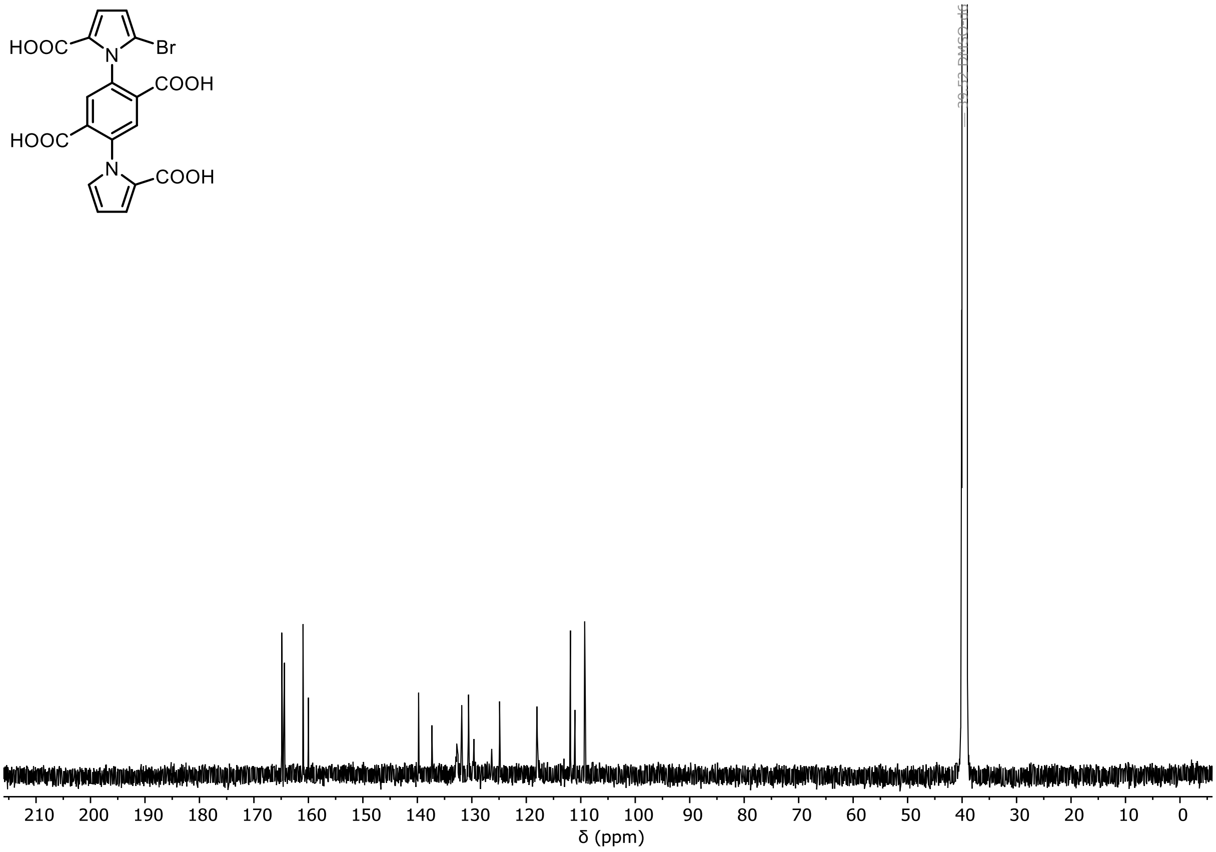

Spectrum S12: ^{13}C NMR spectrum (151 MHz, $(CD_3)_2SO$) of **2b**.

S11. References

S1 Babudri, F.; Fiandanese, V.; Marchese, G.; Punzi, A. A general and straightforward approach to α,ω-ketoesters. *Tetrahedron* **1996**, *52*, 13513–13520.

S2 Nakamura, M.; Gon, M.; Natsuda, S.; Tamai, Y.; Ohkita, H.; Tanaka, K.; Chujo, Y. Development of NIR emissive fully-fused bisboron complexes with π-conjugated systems including multiple azo groups. *Dalton Trans.* **2022**, *51*, 74–84.

S3 Watterson, S. H.; Chen, P.; Zhao, Y.; Dhar, T. G. M; Xiao, Z.; Ballentine, S. K.; Shen, Z.; Fleener, C. A.; Rouleau, K. A.; Obermeier, M.; Yang, Z.; McIntyre, K. W.; Shuster, D.J.; Witmer, M.; Dambach, D.; Chao, S.; Mathur, A.; Chen, B.-C.; Barrish, J. C.; Robl, J. A.; Townsend, I. E. J. Acridone-based inhibitors of inosine 5'-monophosphate dehydrogenase: discovery and sar leading to the identification of *N*-(2-(6-(4-ethylpiperazin-1-yl)pyridin-3-yl)propan-2-yl)-2- fluoro-9-oxo-9,10-dihydroacridine-3-carboxamide (BMS-566419). *J. Med. Chem.* **2007**, *50*, 3730–3742.

S4 Xie, M.-S.; Huang, B.; Li, N.;Tian, Y.; Wu, X.-X.; Deng, Y.; Qu, G.-R.; Guo, H.-M.; Rational design of 2-substituted DMAP-*N*-oxides as acyl transfer catalysts: dynamic kinetic resolution of azlactones. *J. Am. Chem. Soc.* **2020**, *142*, 19226–19238.

S5 Other extra futile cycles are present when racemization of **2b'** is slow compared to the overall fueling process. In this case, the maximal e.e. reached by **2b** via chemostatic fuelling will remain the same as in a fast racemization regime, however, the inherent futile cycles (see Figure S7, C and D) will be overexpressed.

S6 Borsley, S.; Kreidt, E.; Leigh, D. A.; Roberts, B. M. W. Autonomous fuelled directional rotation about a covalent single bond. *Nature* **2022**, *604*, 80–85.

S7 H.-K. Liu; T. W. Mrad; A. Troncossi; S. Borsley; B. M. W. Roberts; A. Betts; D. A. Leigh. Structural influence of the chemical fueling system on a catalysis-driven rotary molecular motor. *J. Am. Chem. Soc.* Accepted (2025).

S8 Rigaku Oxford Diffraction, CrysAlisPro Software system, version 1.171.43.116a, Rigaku Corporation, Wroclaw, Poland, **2024.**

S9 Sheldrick, G.M. SHELXT – Integrated space-group and crystal structure determination. *Acta Cryst.*, **2015**, *A71*, 3–8.

S10Dolomanov, O.V.; Bourhis, L.J.; Gildea, R.J.; Howard, J.A.K.; Puschmann, H. *OLEX2*: a complete structure solution, refinement and analysis program. *J. Appl. Cryst.* **2009**, *42*, 339–341.
